# Choice of anesthesia and data analysis method strongly increases sensitivity of ^18^F-FDG PET imaging during experimental epileptogenesis

**DOI:** 10.1371/journal.pone.0260482

**Published:** 2021-11-24

**Authors:** Ina Jahreis, Pablo Bascuñana, Tobias L. Ross, Jens P. Bankstahl, Marion Bankstahl

**Affiliations:** 1 Department of Nuclear Medicine, Hannover Medical School, Hannover, Germany; 2 Department of Pharmacology, Toxicology and Pharmacy, University of Veterinary Medicine, Hannover, Germany; IRCCS Ospedale Policlinico San Martino, Genova, Italy, ITALY

## Abstract

**Purpose:**

Alterations in brain glucose metabolism detected by 2-deoxy-2-[^18^F]-fluoro-D-glucose (^18^F-FDG) positron emission tomography (PET) may serve as an early predictive biomarker and treatment target for epileptogenesis. Here, we aimed to investigate changes in cerebral glucose metabolism before induction of epileptogenesis, during epileptogenesis as well as during chronic epilepsy. As anesthesia is usually unavoidable for preclinical PET imaging and influences the distribution of the radiotracer, four different protocols were compared.

**Procedures:**

We investigated ^18^F-FDG uptake phase in conscious rats followed by a static scan as well as dynamic scans under continuous isoflurane, medetomidine-midazolam-fentanyl (MMF), or propofol anesthesia. Furthermore, we applied different analysis approaches: atlas-based regional analysis, statistical parametric mapping, and kinetic analysis.

**Results:**

At baseline and compared to uptake in conscious rats, isoflurane and propofol anesthesia resulted in decreased cortical ^18^F-FDG uptake while MMF anesthesia led to a globally decreased tracer uptake. During epileptogenesis, MMF anesthesia was clearly best distinctive for visualization of prominently increased glucometabolism in epilepsy-related brain areas. Kinetic modeling further increased sensitivity, particularly for continuous isoflurane anesthesia. During chronic epilepsy, hypometabolism affecting more or less the whole brain was detectable with all protocols.

**Conclusion:**

This study reveals evaluation of anesthesia protocols for preclinical ^18^F-FDG PET imaging as a critical step in the study design. Together with an appropriate data analysis workflow, the chosen anesthesia protocol may uncover otherwise concealed disease-associated regional glucometabolic changes.

## Introduction

With approximately 50 million affected people worldwide, epilepsy is one of the most common chronic neurological diseases [1]. It is characterized by an excessive neuronal network activity leading to the generation of spontaneous recurrent seizures [2]. Detection of the radiolabeled glucose analogue 2-deoxy-2-[^18^F]-fluoro-D-glucose (^18^F-FDG) by positron emission tomography (PET) is well established to image *in vivo* brain glucose utilization in clinical and preclinical studies [3, 4]. Using this method, focal interictal glucose hypometabolism is a widely detectable phenomenon in chronic epileptic patients [4]. Whereas regional glucose hypometabolism is indicative of reduced metabolic cellular activity e.g. due to neuronal cell death, hypermetabolism is often present in tumor or inflammatory cells [5–7]. A strong glucose hypermetabolism due to exaggerated neuronal network activity during status epilepticus (SE) and single seizures is also detectable in animal models and epileptic patients [8–11]. Alterations in brain glucose metabolism detected by ^18^F-FDG PET may serve as an early predictive biomarker and treatment target for post-brain insult epileptogenesis [12, 13].

Animal models like the lithium-pilocarpine post-SE rat model mimic temporal lobe epilepsy development following an initial brain insult. In contrast to patients where the phase between a brain insult and the potential development of chronic seizures is difficult to study, these models can be used to investigate metabolic brain changes during epileptogenesis by ^18^F-FDG PET [14]. Some studies indicated a decreased glucose metabolism mainly in epilepsy-related areas during the acute or subacute phase post SE, later returning to baseline uptake before showing sometimes hypometabolism at the chronic state [15–20]. Because early alterations in glucose utilization have a potential use as a biomarker and as a treatment target [3, 6], it is important to better understand ^18^F-FDG distribution for longitudinal PET studies. Unfortunately, the published results are not always conclusive especially regarding the affected brain regions, the time point of returning to normal uptake during the subacute phase or detecting hypometabolism in the chronic phase. This might be caused by the use of different image acquisition protocols including different types of anesthesia applied to immobilize the animals at least for the duration of the scan [3, 21]. For example, two recent publications described distinct differences in the time course of hypometabolism after status epilepticus in two rat models of epileptogenesis [18, 19]. Nevertheless, it remains unclear if the reported differences are caused by the different anesthesia protocols (continuous isoflurane vs. awake uptake phase) or by differences between the two animal models.

It is well known that anesthesia can change cerebral blood flow and regional brain metabolism [22, 23], also influencing ^18^F-FDG brain uptake. Nevertheless, general anesthesia established for simultaneous injection of radiotracer and start of a dynamic PET scan is a prerequisite for kinetic modeling which provides additional information about different parameters contributing to the ^18^F-FDG signal, but there is no direct comparison of different anesthesia protocols in animal models of epiletogenesis. Thus, we aimed to analyze changes in brain distribution of ^18^F-FDG for commonly used anesthesia protocols in healthy rats, rats during epileptogenesis and the chronic phase of epilepsy. Our main goal was the identification of a general anesthesia protocol allowing for kinetic analysis of ^18^F-FDG PET data on the one hand and enabling identification of regional metabolic changes on the other hand. Therefore, we applied three continuous anesthesia protocols that are well-tunable or antagonizable, i.e. administration of isoflurane, propofol, or a combination of medetomidine, midazolam and fentanyl. For comparison, we scanned the animals after ^18^F-FDG uptake under awake condition, which was most often used in published studies.

## Material and methods

### Animals

Forty-four female Sprague-Dawley rats were purchased from Envigo Netherlands (Horst, Netherlands) at an age of 9 weeks (body weight of 200 to 220 g) and randomly allocated to experimental groups. They were kept in groups of two rats in individually ventilated cages (Allentown, Neuss, Germany) under controlled climate conditions (20–22°C, 45 to 55% humidity) and a 14/10 h light/dark circle. Standard laboratory chow (Altromin 1234, Lage, Germany) and autoclaved tab water were provided *ad libitum*. All rats were allowed to adapt to the new housing conditions and to repetitive handling for at least one week prior to the start of the experiments. All applicable institutional and/or national guidelines for the care and use of animals were followed. Experimental protocols, including expected mortality and humane endpoints, were approved by the respective agency (Landesamt für Verbraucherschutz und Lebensmittelsicherheit) under animal licence number 14–1441. Experiments were reported according to ARRIVE (Animal Research: Reporting in Vivo Experiments) guidelines [24].

### General experimental design

**Fig 1:**
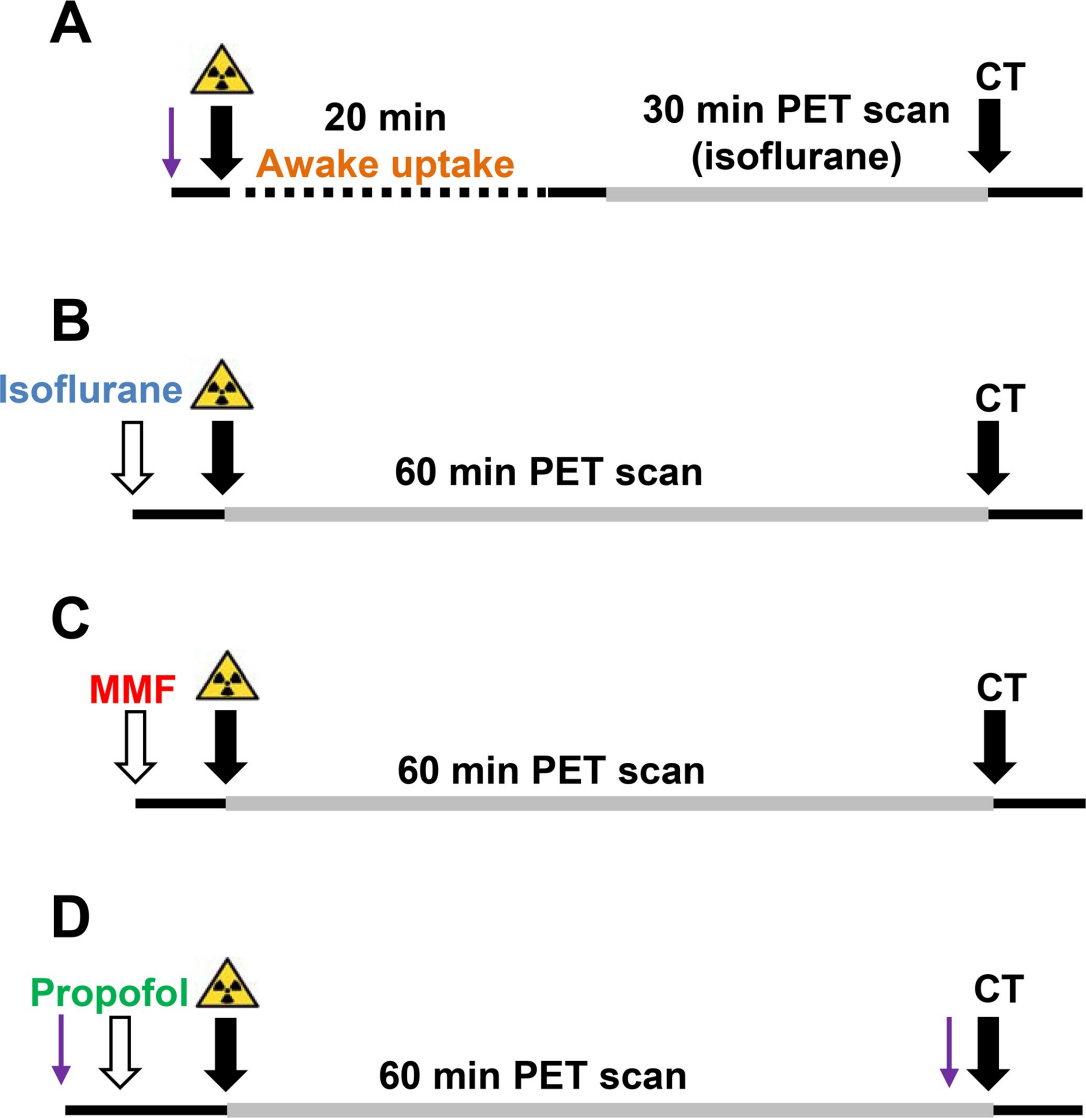
Schematic design of anesthesia protocols illustrating ^18^F-FDG PET scans under (A) awake uptake condition (BL condition n = 11, 7 days after SE n = 11, chronic epileptic timepoint n = 7), (B) continuous isoflurane (BL condition n = 7, 7 days after SE n = 4, chronic epileptic timepoint n = 7), (C) medetomidine-midazolam-fentanyl (MMF, n = 7 for all scanning timepoints) and (D) propofol anesthesia (BL condition n = 8, 7 days after SE n = 7, chronic epileptic timepoint n = 7). Arrows indicate start of anesthesia induction (white), start of PET or CT scans (black), or start of a short additional isoflurane anesthesia (purple).

The lithium-pilocarpine post-SE rat model was used to induce epileptogenesis in the animals. Before SE induction a baseline ^18^F-FDG PET scan was performed, followed by a PET scan at 7 days post SE and one in the phase of chronic epilepsy between 12 to 14 weeks post SE. The time period for investigating chronic epilepsy is way beyond the onset of spontaneous seizures in pilocarpine post-SE rat models when the disease is stabilized [25]. For logistical reasons, not all animals of a single group were scanned at each time point. Information about group sizes at the single imaging time points can be found in S1 Table. For each of the three scanning timepoints, four different anesthesia protocols were tested (Fig 1): ^18^F-FDG uptake in awake rats followed by a 30 minutes PET scan from minute 30 to 60 after tracer injection, or continuous anesthesia for a 60 minutes dynamic PET scan under either isoflurane, medetomidine-midazolam-fentanyl (MMF) or propofol anesthesia. Blood glucose levels were measured each time before tracer injection and after the CT scan. For all rats scanned at the chronic timepoint, at least one spontaneous generalized epileptic seizure being observed during presence of experimenters in the animal housing room was recorded.

### Induction of SE

SE was induced in 38 rats by fractionated pilocarpine injection as described previously, which was applied in order to reduce SE-associated mortality [26]. Briefly, animals were pre-treated with lithium chloride (127 mg/kg p.o.; Sigma-Aldrich, Steinheim, Germany) approximately 16 h prior to the first pilocarpine injection. To reduce parasympathic side effects, methyl-scopolamine (1 mg/kg i.p., Sigma-Aldrich) was administered 30 minutes before a bolus injection of pilocarpine (30 mg/kg i.p., Sigma-Aldrich). If no seizure activities were shown, rats were administered a maximum of 3 further injections (10 mg/kg i.p. per injection). Seizure activity was monitored by two trained persons. Onset of SE was marked by repetitive generalized convulsive, stage 4 (rearing) and 5 (rearing and falling) seizures according to Racine’s scale [27]. After 37.63 ± 7.76 mg/kg pilocarpine all 38 rats developed a self-sustaining SE. Ninety minutes after SE onset, rats were treated with two injections of diazepam (10 mg/kg in 2 ml/kg per injection, Ratiopharm, Ulm, Germany) given at intervals of 15 to 20 minutes. A third dose of diazepam (5 mg/kg) was administered if motor seizure activity was still present. Additionally, rats received a subcutaneous injection of 5 ml glucose electrolyte solution (Sterofundin HEG-5, B. Braun, Melsungen, Germany) to keep them hydrated and rats were placed on heating pads to prevent hypothermia. For the first week following SE, rats were weighted daily, mashed laboratory chow was offered and, if needed, rats were hand-fed several times per day. Despite these measures 6 rats died spontaneously between 24 and 48 hours after SE induction by unknown cause. Regular monitoring of these animals did not reveal any sign of seizure activity or excessive weight loss that could explain these deaths. One animal was killed by an overdose of an anesthetic due to reaching an end point criterion (weight loss), resulting in a total mortality of 18.42% (7/38 rats).

### Anesthesia and scanning protocols

A dedicated small animal PET scanner (Inveon DPET, Siemens Knoxville, TN, USA) was used for PET imaging. ^18^F-FDG PET scans were performed at baseline, during epileptogenesis at 7 d post SE and in the chronic phase of epilepsy at 12 to 14 weeks post SE. Animals were monitored for at least 60 minutes before each scan to exclude occurrence spontaneous seizures during this period. Directly after induction of anesthesia before radiotracer injection and after the CT scan, blood glucose levels were measured by a micropuncture of the saphenous vein (Conrour XT®, Bayer Consumer Care, Basel, Schweiz). An overall amount of 19.66 ± 1.99 MBq ^18^F-FDG in 0.3 ml saline was injected via a lateral tail vein. Anesthetized animals were placed in dedicated animal beds (Minerve, Esternay, France), eyes were protected from drying out with a dexpanthenol-containing eye ointment (Bepanthen® Nasen- und Augensalbe, Bayer AG, Leverkusen, Germany) and respiration rate (BioVet software, m2m Imaging, Cleveland, OH, USA) was used to monitor depth of anesthesia. During the whole imaging procedure animals were warmed to avoid hypothermia. Acquired images were reconstructed by an iterative OSEM3D/fastMAP (ordered subset expectation maximization 3-dimensional/maximum a posteriori) algorithm including corrections for decay, attenuation, random events, and scatter. For attenuation correction, standard 20 minutes ^57^Co transmission scans were used, separately performed in individuals of similar weight with identical positioning in the scanner. A fast low-dose CT scan (Inveon CT, Siemens) was performed to facilitate co-registration afterwards. For awake uptake (Fig 1A), ^18^F-FDG was injected under a short isoflurane (Baxter Unterschleißheim, Germany) anesthesia. After a radiotracer uptake phase of 20 minutes under awake condition, rats were again anesthetized with isoflurane (3% for induction, 1.0–3.0% for maintenance). A static scan was conducted from 30 to 60 minutes after ^18^F-FDG injection with the brain in the center of the field of view. Eleven rats were scanned for BL, 11 at 7 d post SE and 7 in the chronic epileptic phase. Regarding the calculation of the uptake, the scanning time frame between 30 to 60 minutes was chosen as it usually provides the needed steady-state considering the time activity curve (TAC) of ^18^F-FDG for the brain [28]. For the further three anesthesia protocols, ^18^F-FDG was injected simultaneously with the start of a dynamic 60 minutes PET scan. For isoflurane anesthesia (Fig 1B), scans were performed under continuous isoflurane anesthesia (3% for induction, 1.0–3.0% for maintenance; resulting in an average respiratory rate of 36.73 ± 4.05 per minute). Seven animals were scanned for BL, 4 at 7 d post SE and 7 at the chronic epileptic timepoint. For the completely antagonizable MMF anesthesia (Fig 1C), animals received an intramuscular injection of 0.15 mg/kg medetomidine (Domitor®, Janssen-Cilag, Neuss, Germany), 2 mg/kg midazolam (Dormicum, Roche Pharma AG, Grenzach-Wyhlen, Germany) and 0.005 mg/kg fentanyl (Fentadon, Albrecht GmbH Aulendorf, Germany) in 1 ml/kg saline. Seven rats were scanned for each timepoint. For propofol (Fig 1D), a catheter was placed in a lateral tail vein during a short isoflurane anesthesia. Through this catheter, a continuous propofol administration (Propofol-®Lipuro, B.Braun Melsungen AG, Melsungen, Germany) with an infusion rate of 45 mg/kg/h (4.5 ml/kg/h) was started via a syringe pump (Model PHD Ultra, Harvard Apparatus Inc., South Natick, Massachusetts, USA) and the isoflurane supply was stopped. After 5 minutes, when the propofol anesthesia was stable, the radiotracer was injected simultaneously to the start of the PET scan. The following CT scan was again performed under isoflurane anesthesia. Eight rats were scanned for the BL and the 7 d post-SE timepoint, while 7 rats underwent scans for the chronic epileptic timepoint. One animal showed unstable anesthesia during the 7-d scan and was therefore excluded from the analysis.

### PET image analyses

PET analysis was performed by an experienced scientist, blinded to the experimental treatment. PET images were fused to a standard T2-weighted MRI rat brain template [29] using PMOD 3.703 fusion tool. To this aim, the CT images were first co-registered to the MRI template and afterwards matched to the corresponding PET images. ^18^F-FDG uptake was calculated as percentage injected dose per cubic centimeter of tissue (%ID/cm^3^). Earlier ^18^F-FDG studies in epilepsy models also used the calculation of the standard uptake value (SUV = (kBq/cm³) / (injected dose / body weight)). However, for long-term epileptogenesis studies the body weight and the body ratio of fat and water changes due to status epilepticus-related weight loss and age-/epilepsy-related weight gain. Thus, SUV calculation can also not be generally recommended, since it might lead to over- and underestimation of the ^18^F-FDG uptake [30]. Regions of interest (ROI) were analyzed by applying a detailed rat brain atlas [31] to the co-registered images. Additionally, different approaches of kinetic analysis were evaluated. Therefore, list-mode data were histogrammed to 32 frames of 5 x 2, 4 x 5, 3 x 10, 8 x 30, 5 x 60, 4 x 300 and 3 x 600 s, respectively. An image derived arterial input function (IDIF) was created by drawing two volumes of interest (VOI, 2x2x4 mm^3^) in both carotid arteries to measure a TAC. Additionally, VOI-atlas-based kinetic analysis using both the 2-tissue compartment model for ^18^F-FDG were applied and the metabolic rate (MR_Glu_) and the influx rate constant (K_i_) of glucose calculated [32]. A lumped constant of 0.71 was assumed [33], and the blood glucose level averaged from both measurements (S1 Fig) was applied.

Additionally, the co-registered uptake images were used for statistical parametric mapping (SPM) analysis, calculated using SPM12 software (UCL, London, UK). SPM was used for the calculation of baseline differences in ^18^F-FDG uptake under awake condition compared to the other three anesthesia protocols. Additionally, BL scans of each anesthesia protocol were compared to scans conducted 7 d or 12 to 14 weeks after SE. Differences were calculated by a two-sample unpaired t-test using SPM12 software. A significance level threshold of 0.05 (uncorrected for multiple comparisons) and a minimum cluster size of 100 voxels were chosen. After each comparison parametric t-maps were loaded in PMOD and significantly-changed voxels were located by co-registration with the MRI template.

### Statistical analysis

All data are presented as mean ± standard deviation (SD). Data was analyzed with GraphPad Prism 7 software (GraphPad, La Jolla, CA, USA). One-way ANOVA followed by Dunnett’s post hoc test was applied for intragroup comparison of blood glucose levels of the first and second measurements. One-way ANOVA followed by Sidak’s multiple comparisons test was performed for comparison of the first and the second blood glucose level measurement of each scanning timepoint. Intergroup differences in averaged blood glucose levels were calculated by one-way ANOVA and Tukey’s post hoc test and intragroup differences by one-way ANOVA followed by Dunnett’s post hoc test. For the comparison of activity signal in amygdala and thalamus derived from TACs and maximum peaks in IDIF between the three continuous anesthesia one-way ANOVA and Dunnett’s post hoc test was used. For statistical analysis of imaging data, one-way ANOVA followed by Dunnett’s post hoc test was used to analyze inter-anesthesia differences in ^18^F-FDG brain uptake at baseline, and intragroup differences in ^18^F-FDG uptake, K_i_, and MR_Glu_ following SE. Group size, based on expected variances and differences, was estimated by Power analysis. ANOVA results are provided as F-ratio, degrees of freedom (DF), and P-value. A P-value < 0.05 was considered statistically significant.

## Results

### Blood glucose levels

**Fig 2:**
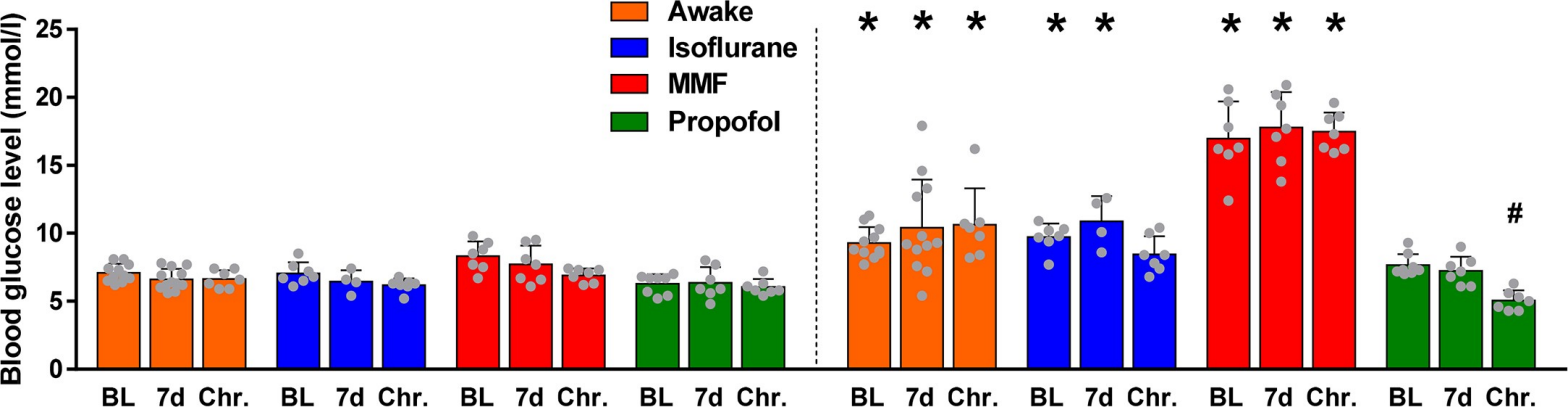
Blood glucose levels measured before ^18^F-FDG injection (left) and after the CT scan (right). Data is presented as mean ± SD. No group differences were detectable comparing first measurements by one-way ANOVA and Dunnett‘s post hoc test, P<0.05. * indicate changes comparing second vs. first measurement (one-way ANOVA, Sidak‘s multiple comparisons test, P<0.05). # indicate changes between second measurements at 7 d post SE or at the chronic epileptic timepoint and BL for each anesthesia (one-way ANOVA, Dunnett‘s post hoc test, P<0.05).

Initial blood glucose levels (baseline) did not differ between groups (Fig 2). Impact of anesthesia protocols on blood glucose levels were evaluated by comparing the first with the second glucose measurement of each PET scan. An increase in blood glucose level was detectable for tracer uptake under awake condition (ANOVA: F-ratio = 38.29, DF = 23, P<0.0001; BL, +29.69%, P = 0.0106; 7 d post SE, +48.62%, P<0.0001; chronic epileptic phase, +60.12%, P<0.0001), under continuous isoflurane anesthesia (BL, +37.65%, P = 0.0153; 7 d post SE, +68.60%, P = 0.007; chronic epileptic phase, +36.72%, P = 0.0664) and most prominently under MMF anesthesia (BL, +103,77%, P<0.0001; 7 d post SE, +129.94%, P<0.0001; chronic epileptic phase, +152.69%, P<0.0001). For propofol anesthesia, no changes were detectable. Intra-anesthesia comparison of second-measurement blood glucose levels revealed a decrease in chronic epileptic rats versus the BL timepoint for propofol anesthesia (ANOVA: F-ratio = 18.65, DF = 2, P<0.0001; -34%, P<0.0001).

### Comparison of anesthesia protocols in healthy rats

**Fig 3:**
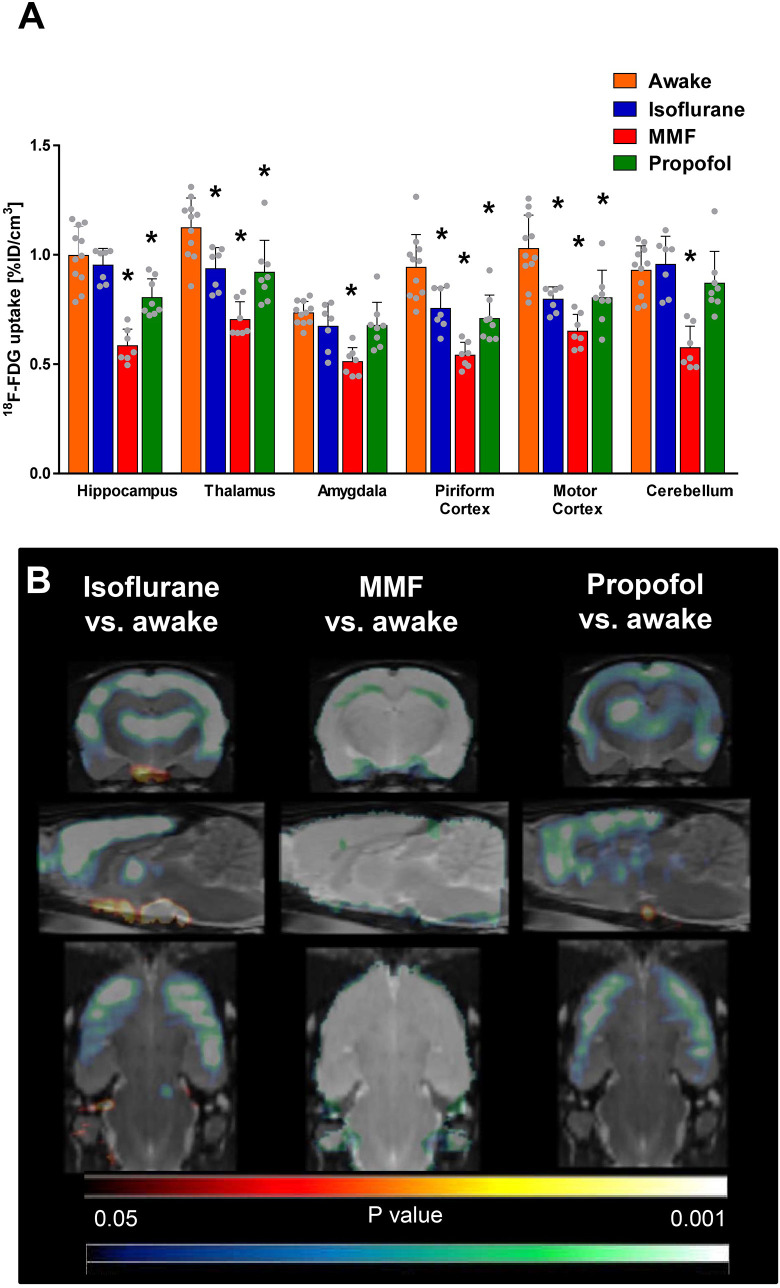
^18^F-FDG brain uptake under BL conditions. (A) Regional data is presented as mean ± SD. * indicate significant differences between tracer uptake under awake conditions and different anesthesia protocols, tested by one-way ANOVA, Dunnett‘s multiple comparison test, P<0.05. (B) Coronal, sagittal and horizontal (-3.6, 0.4, -8.1 mm relative to bregma) t-maps resulting from voxel-wise comparisons (statistical parametric mapping, SPM) of ^18^F-FDG uptake in awake rats and under continuous isoflurane (left), MMF (middle) or propofol anesthesia (right). Only clusters with significantly different voxels are shown (Student’s t test, P<0.05, minimum cluster size of 100 voxels). Cold scale represents significantly decreased P-values, hot scale significantly increased P-values for each voxel.

All anesthesia protocols were feasible for the imaging studies and well tolerated. Compared to PET scans performed after awake tracer uptake condition, continuous isoflurane anesthesia resulted in significantly lower ^18^F-FDG uptake in cortical regions, like piriform cortex (ANOVA: F-ratio = 18.32, DF = 3, P<0.0001; -20.21%, P = 0.0097) or motor cortex (ANOVA: F-ratio = 15.94, DF = 3, P<0.0001; -22.33%, P = 0.0016), and in thalamus (ANOVA: F-ratio = 17.12, DF = 3, P<0.0001; -16.07%, P = 0.0059, Fig 3A). SPM analysis confirmed this finding (Fig 3B). No group differences were detected for the hippocampus, the amygdala, and the cerebellum.

^18^F-FDG uptake under MMF anesthesia was significantly lower in all analyzed brain areas and showed uptake values 30% to 43% below those for tracer uptake in conscious rats (Fig 3A; piriform cortex, -42.55%, P<0.0001; motor cortex -36.89%, P<0.0001; hippocampus, ANOVA: F-ratio = 26.63, DF = 3, P<0.0001; -42.00%, P<0.0001; thalamus, -37.50%, P<0.0001; amygdala, ANOVA: F-ratio = 10.62, DF = 3, P<0.0001; -30.14%, P<0.0001; cerebellum, ANOVA: F-ratio = 15.08, DF = 3, P<0.0001; -38.71%, P<0.0001). SPM analysis revealed a globally lower tracer uptake in the whole brain (Fig 3B).

Propofol anesthesia resulted in a similar pattern to isoflurane anesthesia compared to tracer uptake in conscious rats both in the regional as well as in the SPM analyses (Fig 3A and 3B). Piriform cortex (-24.47%, P = 0.0016), motor cortex (-6.91%, P = 0.0035), hippocampus (-20.00%, P = 0.0023) and thalamus (-17.86%, P = 0.0061) showed significantly less ^18^F-FDG uptake. In amygdala and cerebellum no significant differences were detectable.

### ^18^F-FDG uptake during epileptogenesis

**Fig 4:**
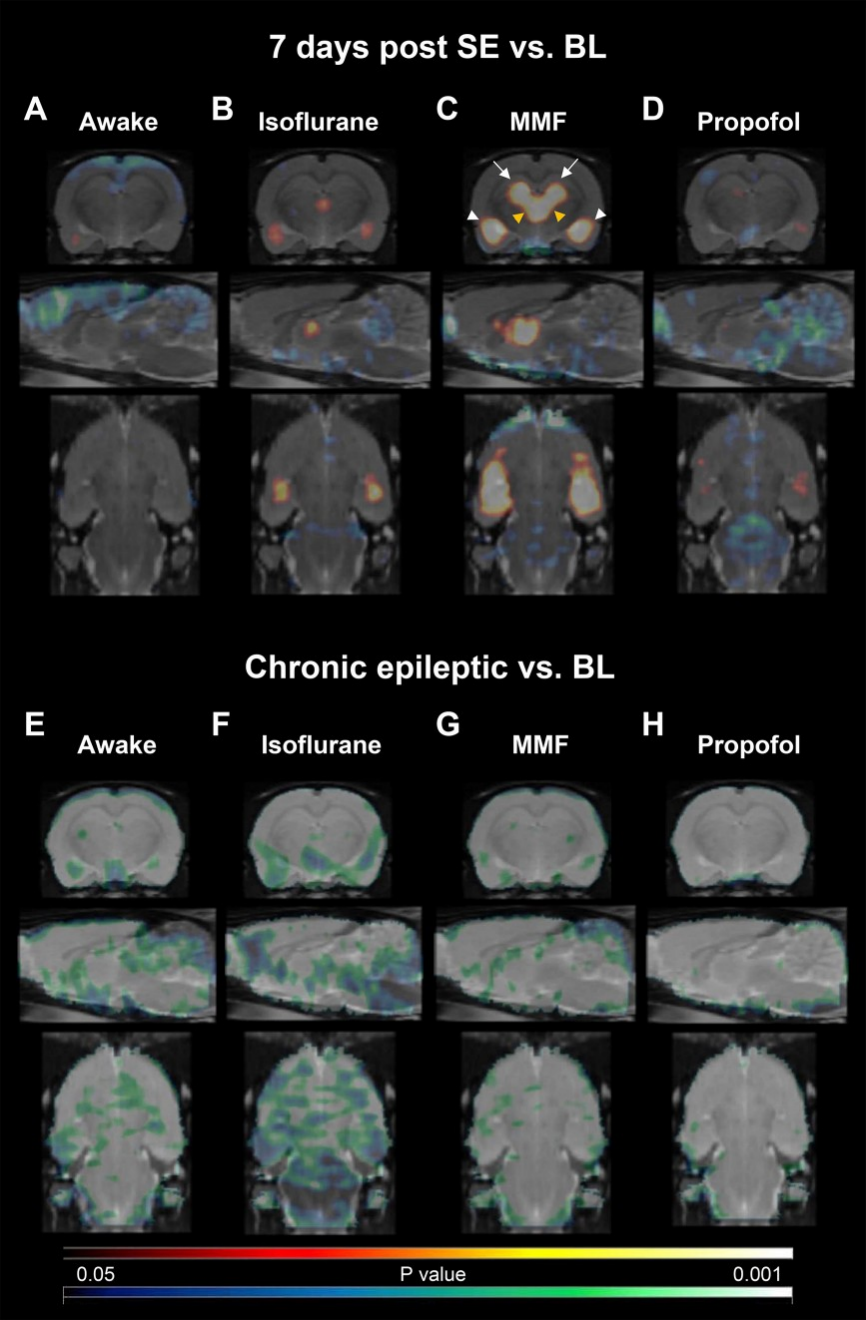
^18^F-FDG uptake during epileptogenesis and chronic epilepsy. Coronal, sagittal and horizontal (-3.6, 0.4, -8.1 mm relative to bregma) SPM t-maps resulting from voxel-wise comparisons of ^18^F-FDG uptake under BL condition compared with uptake in (A-D) 7 d post SE or (E-H) chronic epileptic rats: (A, E) awake, (B, F) isoflurane, (C, G) MMF and (D, H) propofol anesthesia. Only clusters with significantly different voxels are shown (Student’s t-test, P<0.05, minimum cluster size of 100 voxels). Cold scale represents significantly decreased P-values, hot scale significantly increased P-values for each voxel. In (C), white arrows point on prominent changes in the hippocampus, white arrowheads to changes in the amygdala/piriform cortex and yellow arrowheads to changes in the dorsal thalamus.

Compared to BL, ^18^F-FDG uptake in conscious rats at 7 d post SE remained unchanged in all analyzed brain regions (S2A and S3B Figs). However, SPM analysis revealed significantly decreased voxels in cortical regions and cerebellum (Fig 4A). For chronic epilepsy, a generalized decrease was detectable both in the SPM (Fig 4E) and the atlas-based analysis (S2A Fig).

Comparing ^18^F-FDG uptake values under continuous isoflurane anesthesia at BL to 7 d post SE, the SPM map detected significantly increased uptake in subregions of the amygdala and thalamus (Fig 4B), whereas regional analysis did not reveal significant changes for amygdala, thalamus, hippocampus, piriform cortex or motor cortex, and cerebellum (S2B Fig). Only pons showed a slight decrease in ^18^F-FDG uptake (ANOVA: F-ratio = 5.862, DF = 2, P = 0.0131; -17.87%, P = 0.0337). In the chronic phase of epilepsy, a globally decreased uptake was detectable in both the SPM (Fig 4F) and the regional analysis being most prominent in hippocampus (ANOVA: F-ratio = 18.22, DF = 2, P<0.0001; -33.96%, P = 0.0001) and least distinct in the pons (-17.83%, P = 0.0137, S2B and S3B Figs).

For MMF anesthesia SPM analysis revealed a distinctly increased uptake in thalamus, amygdala and hippocampus at 7 d post SE (Fig 4C). Regional analysis (S2C Fig) showed significantly increased values only for thalamus (ANOVA: F-ratio = 25.23, DF = 2, P<0.0001; +19.15%, P = 0.0266) and amygdala (ANOVA: F-ratio = 28.77, DF = 2, P<0.0001; +21.47%, P = 0.0133) while no changes were detectable for hippocampus, piriform cortex, motor cortex, pons and cerebellum. Uptake in the phase of chronic epilepsy was decreased in the whole brain in the SPM analysis (Fig 4F) ranging between 37.40% reduction in hippocampus (ANOVA: F-ratio = 25.41, DF = 2, P<0.0001; P = 0.0003) and 27.61% reduction in pons (ANOVA: F-ratio = 15.22, DF = 2, P<0.0001; P<0.0001) in the regional analysis (S2C and S3C Figs).

Comparing ^18^F-FDG uptake under propofol anesthesia at BL with 7 d post SE, a decreased uptake was detectable only for pons (ANOVA: F-ratio = 11.51, DF = 2, P = 0.0005; -16.31%, P = 0.0439, S2D Fig). This was also clearly seen in the SPM analysis (Fig 4D), while scattered decreased uptake became visible in cerebellum and cortical areas as well as a limited increased uptake in the amygdala. Comparing uptake at the chronic epileptic timepoint to BL, a generally decreased uptake was identified in the SPM analysis (Fig 4H) ranging from 45.84% reduction in hippocampus (ANOVA: F-ratio = 34.62, DF = 2, P<0.0001; P<0.0001) to 31.88% reduction in pons (P = 0.0003, S2D and S3D Figs) in the regional analysis.

### Time activity curves and image-derived input function

**Fig 5:**
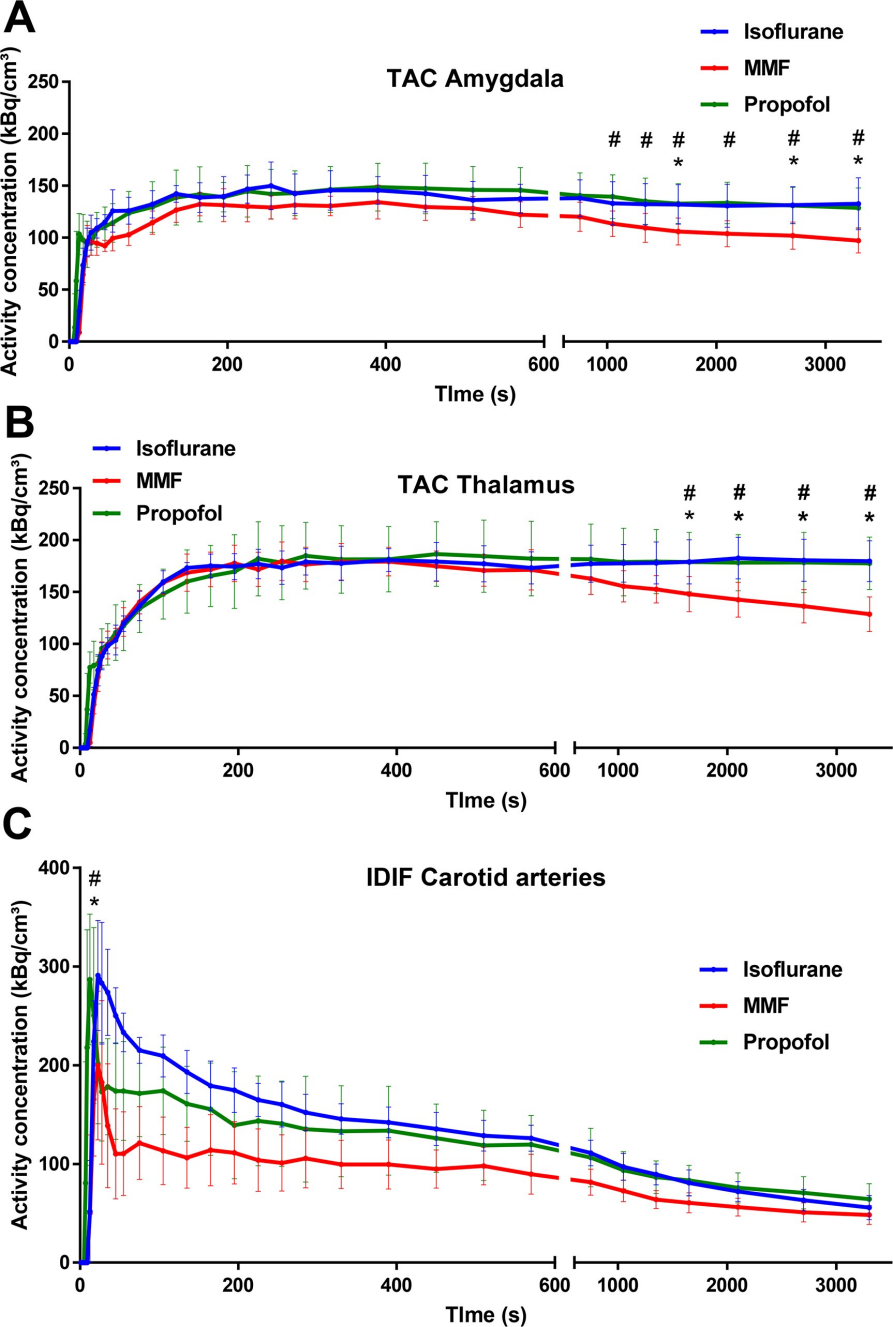
Representative time activity curves for (A) amygdala, (B) thalamus and (C) image derived input function (IDIF, carotid arteries) for BL scans conducted under isoflurane, MMF and propofol anesthesia. Data is presented as mean ± SD. * indicate significant differences between MMF and isoflurane anesthesia and # between MMF and propofol anesthesia calculated by one-way ANOVA and Dunnett’s post hoc test.

Under all three anesthesia conditions, the ^18^F-FDG TACs for brain regions like amygdala or thalamus (Fig 5A and 5B) reached an activity plateau after approximately 195 seconds which remained stable for the whole scanning period using isoflurane and propofol anesthesia. However, the TAC for MMF anesthesia started to decline about 510 seconds after injection and was lower compared to propofol anesthesia from 17.5 minutes (amygdala) or 27.5 minutes (thalamus) on (Fig 5A and 5B). For the last scanning frame, the activity concentration in amygdala under MMF anesthesia was 32.16% lower compared to propofol anesthesia (ANOVA: F-ratio = 6.976, DF = 2, P = 0.0053; P = 0.0111) and 36.26% lower compared to isoflurane anesthesia (P = 0.0059; Fig 5A). Likewise, the activity concentration in thalamus under MMF anesthesia for the last frame was 38.09% lower compared to propofol anesthesia (ANOVA: F-ratio = 13.4, DF = 2, P = 0.0002; P = 0.0005) and 39.75% lower compared to isoflurane anesthesia (P = 0.0005, Fig 5B).

For obtaining an IDIF from the blood pool, we used the left ventricle, the vena cava, or carotid arteries (Fig 5C) for drawing the ROIs. Only the IDIF derived from the carotid arteries turned out to be reliable. By averaging the values from IDIF obtained from both carotid arteries for 60 minutes BL scans, the maximum peak under MMF anesthesia was lower compared to isoflurane anesthesia (ANOVA: F-ratio = 4.29, DF = 2, P = 0.0290; -45.54%, P = 0.0345) and propofol anesthesia (-43.47%, P = 0.0374, Fig 5C). At the end of the scan, the IDIFs for all three anesthesia protocols reached similar values without significant differences.

### Kinetic analysis

We used the IDIF derived from carotid arteries of each animal and the averaged blood glucose level (S1 Fig) and the 2-tissue compartment model for FDG for region-based kinetic analysis.

**Fig 6:**
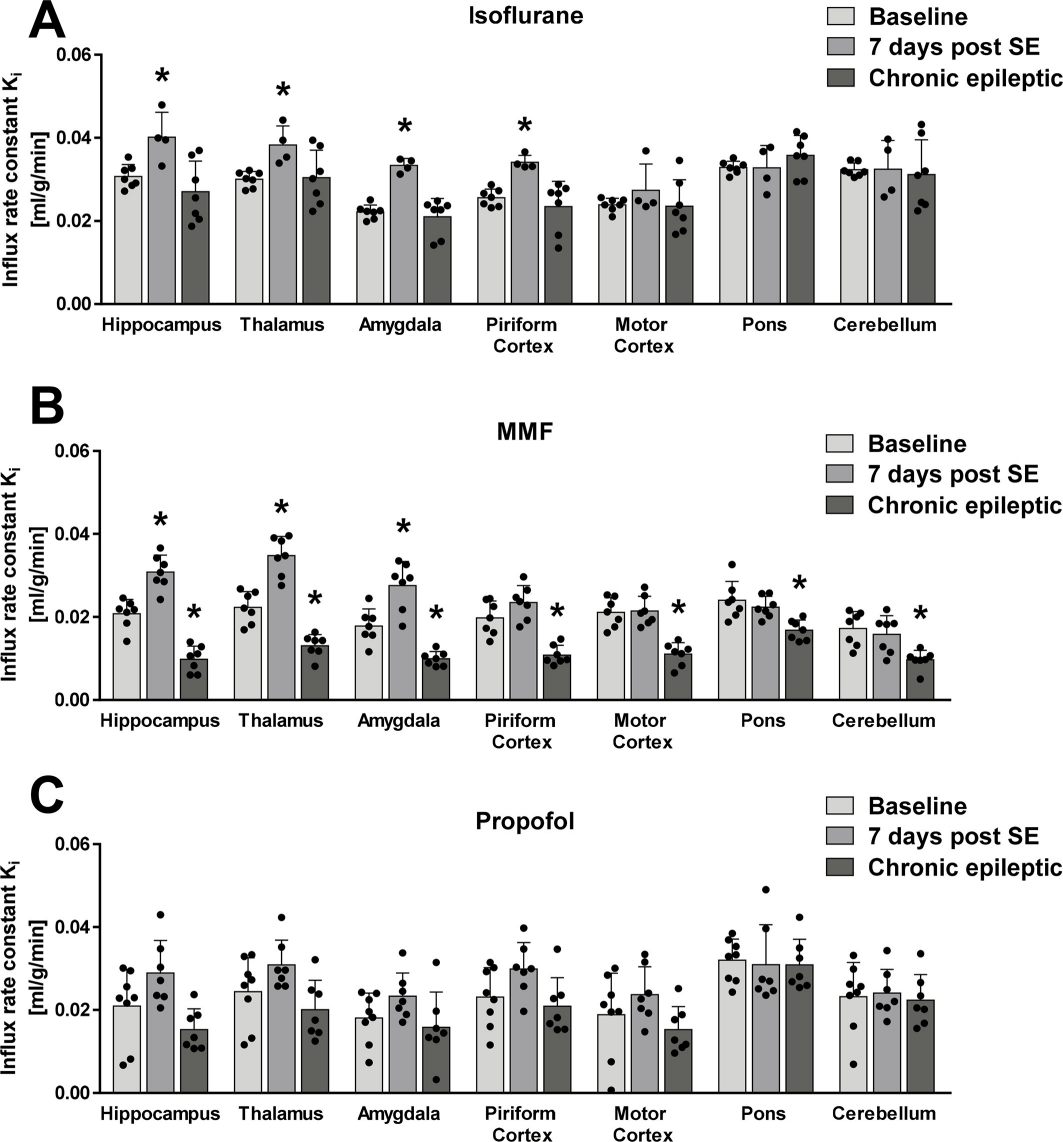
Calculation of the influx rate constant K_i_ of ^18^F-FDG by a 2-tissue compartment model for (A) continuous isoflurane, (B) MMF and (C) propofol anesthesia. Data is presented as mean ± SD. Significant changes in brain regions between BL and following scans were tested by one-way ANOVA and Dunnett’s multiple comparisons test. * indicate significant differences (P<0.05).

Thus, the IDIF-based K_i_ for BL to 7 d post-SE scans under isoflurane anesthesia revealed significant increases for hippocampus (ANOVA: F-ratio = 6.884, DF = 2, P = 0.0076; +30.70%, P = 0.0333), thalamus (ANOVA: F-ratio = 4.342, DF = 2, P = 0.0325; +27.37%, P = 0.0288), amygdala (ANOVA: F-ratio = 22.81, DF = 2, P<0.0001; +50.47%, P<0.0001) and piriform cortex (ANOVA: F-ratio = 8.941, DF = 23, P = 0.0028; +33.28%, P = 0.0088, Fig 6A). Comparing BL vs. scans conducted at the chronic epileptic timepoint, no brain region showed a significantly changed K_i_ (Fig 6A). Using MMF anesthesia, K_i_ for the scan 7 d post SE was increased for hippocampus (ANOVA: F-ratio = 59.7, DF = 2, P<0.0001; +48.56%, P = 0.0001), thalamus (ANOVA: F-ratio = 56.36, DF = 2, P<0.0001; +55.83%, P<0.0001) and amygdala (ANOVA: F-ratio = 31.12, DF = 2, P<0.0001; +54.62%, P = 0.0008) compared to BL scans, while K_i_ remained unchanged for piriform and motor cortex, pons or cerebellum (Fig 6B). However, significantly decreased K_i_ values were detectable for all brain regions ranging from 47.43% reduction in the hippocampus (P<0.0001) to 29.93% reduction in pons (P = 0.0015, Fig 6B) at the chronic epileptic timepoint compared to BL. For propofol anesthesia, K_i_ was not significantly changed for both 7 d post SE and chronic epilepsy compared to BL scans (Fig 6C). Calculation of IDIF-based MR_Glu_ (S4 Fig) resulted in comparable results to IDIF-based K_i_ (Fig 6).

## Discussion

Our study focused on comparing cerebral ^18^F-FDG distribution following epileptogenesis induction using different anesthesia protocols for static and dynamic PET scans to identify the most sensitive scanning and analysis protocols. Our major findings include first a prominent regional glucose hypermetabolism during epileptogenesis, best detectable under MMF anesthesia. Second, this anesthesia protocol was more sensitive in revealing lower tracer uptake than scanning animals after ^18^F-FDG uptake in conscious rats, which is considered to be most translational to scans in human patients [28]. Third, kinetic modeling improved sensitivity for detecting increased glucose turnover when using continuous isoflurane supply as standard anesthesia.

Undoubtedly, scanning of animals being conscious during the whole imaging procedure would be the most comparable approach to clinical ^18^F-FDG PET. While some studies performed imaging with continuously awake, restrained animals or partly unrestrained rats using motion correction, findings may still reflect a stressed condition of the animals, and gaining blood samples or drawing ROIs for input functions is difficult [21, 28, 34]. A recent study has shown that it is possible to overcome these limitations [35]. Nevertheless, this approach is challenging as a standard method and not broadly available. Alternatively, scanning after a tracer uptake phase under the awake condition is supposed to be a feasible, still well translatable approach [22]. In our study, we decided to use a very short isoflurane anesthesia for tracer administration before the awake uptake in order to limit the stress for the animals. We cannot exclude that this influenced the awake uptake phase to a certain degree. One of the problems, however, is that such a protocol does also not allow the acquisition of dynamic scans which might uncover further important epilepsy-related changes. Thus, we here used well-established, antagonizable or adjustable, anesthesia protocols, i.e. administering isoflurane, MMF and propofol, for acquiring dynamic scans. Although isoflurane is known to influence brain perfusion, inhalation anesthesia is the method of choice for imaging rodents [36]. The combined and completely antagonizable MMF anesthesia consisting of the benzodiazepine midazolam, the α_2_-adrenergic agonist medetomidine, and the opioid fentanyl was shown to be suitable for sustained/enduring anesthesia in rats despite associated impact on blood pressure and heart rate [37, 38]. Propofol, partially mediating its anesthetic effects via enhancing the function of GABA-activated chloride channels, induces a decreased cerebral blood flow and perfusion pressure, which may affect tracer brain uptake [21, 36, 39]. However, due to propofol’s rapid metabolization and elimination from the body the anesthesia is well controllable and thus also commonly used for anesthetizing rodents for imaging procedures [36]. In addition to the influence on the circulation, the effect of anesthesia on blood glucose levels is relevant for investigations using ^18^F FDG PET, since blood glucose competes with ^18^F-FDG for being transported by glucose transporters at blood-tissue and extra-intracellular barriers.

In this study, we show that basal blood glucose levels are not affected by the SE or by epilepsy development itself. However, while all anesthesia protocols were well tolerated and practical for PET imaging, isoflurane and especially MMF anesthesia led to increased blood glucose levels. While isoflurane impairs glucose-stimulated insulin release leading to reduced cellular glucose uptake from the blood [40] the even more prominent increase of blood glucose levels during MMF anesthesia can be explained by medetomidine-induced inhibition of pancreatic beta-cell’s insulin secretion [41–44]. Subsequent compensatory increase in hepatic glucose generation and release as well as potential fentanyl-mediated inhibition of insulin secretion, which has been demonstrated in vitro [45], probably additionally contributed to the effect.

By influencing brain activity, circulation and blood glucose, which shares the same metabolic route as ^18^F-FDG and therefore competes for transport and phosphorylation, anesthetics have also an impact on ^18^F-FDG uptake values [21, 46]. In healthy rats (baseline), isoflurane and propofol anesthesia resulted in suppressed cortical and thalamic uptake. This pattern was probably induced by their action on GABAergic neurons, whose density is high in these brain regions [22, 47]. MMF showed generally decreased uptake, which might have additionally been caused by the high blood glucose levels.

A restricted amount of decreased voxels was detectable in cortical areas after ^18^F-FDG uptake in conscious rats comparing 7 d post-SE to BL scans. This detection might be possible because in contrast to the conscious uptake condition (Fig 3) each further investigated anesthesia suppressed cortical ^18^F-FDG uptake in healthy rats. Additionally, for all anesthesia increased voxels in amygdala were detected by SPM analysis. However, only with MMF anesthesia this increase was rather prominent and became statistically significant using atlas-based regional analysis, which might be due to the generally lower ^18^F-FDG uptake under BL conditions. In chronic epileptic animals, a hypometabolism was detectable by all investigated anesthesia. Interestingly, the high sensitivity of MMF anesthesia for detection of differences using brain ^18^F-FDG PET was also described for auditory stimulation paradigms in the Mongolian gerbil [48]. Compared to recent studies in the lithium-pilocarpine rat model under the awake uptake condition, we found decreased ^18^F-FDG uptake mainly in cortical regions but did not detect the wide-spread glucose hypometabolism in epilepsy-related areas at 3 days post SE [15, 16, 18, 49] or at 7 days post SE [16, 17]. Pons or whole brain was used as a reference region for normalization of ^18^F-FDG uptake in most of these studies. Particularly pons is assumed to be less affected by epileptogenesis [10] and is therefore used as internal reference region [16, 18, 50]. However, in the present study both pons and cerebellum (another commonly applied reference region) revealed decreased tracer uptake compared to BL during epileptogenesis or chronic epilepsy (S2 Fig) and were therefore not used as a reference for ^18^F-FDG turnover.

Although alternative simplified approaches exist to circumvent the dynamic acquisition [51], kinetic modeling is considered the gold standard for brain PET data analysis. Kinetic modeling for the ^18^F-FDG 2-tissue compartment model requires the acquisition of an arterial input function. Ideally, an input function is derived from repeated arterial blood samples. However, in multiple-scan longitudinal PET studies using animals weakened by a disease state like SE extensive blood sampling is not feasible. Therefore, we here tried instead to generate IDIFs by drawing VOIs in the cava vein or the left ventricle as recently described [52, 53]. This approach was not feasible for this study due to spill-in. Thus, IDIFs from carotid arteries were used for the kinetic modeling. However, also these input functions showed moderate disturbances caused by spill-in from the surrounding tissue as there was still activity detected in the last scanning frames. The IDIF for MMF remained below values reached under isoflurane or propofol anesthesia, indicating a rapid redistribution of the tracer. In line with this result, the TACs for representative brain regions showed that the steady-state of ^18^F-FDG accumulation in the brain only lasted for less than 10 minutes under MMF anesthesia. In the following, we used the 2-tissue compartment model for ^18^F-FDG. For comparing anesthesia, we mainly focused on the values for IDIF-based K_i_, because they were not additionally corrected for blood glucose levels. Nevertheless, the pattern of changed values for IDIF-based K_i_ was similar to that for IDIF-based MR_Glu_. For K_i_, significant increase was detected for epilepsy-associated brain areas like hippocampus, thalamus and amygdala both with isoflurane and MMF anesthesia. This aligned generally well with the findings using SPM analysis for the ^18^F-FDG uptake, but was of added value in terms of higher sensitivity for regionally increased glucose turnover during epileptogenesis especially with the standard anesthesia protocol (continuous isoflurane).

So far, the causes underlying brain glucometabolic changes are only poorly understood [54]. Hippocampal cell loss and atrophy, altered expression of glucose transporters, changed cerebral blood flow, neuroinflammation and reorganization, or altered synaptic transmission might contribute to changes in glucose metabolism [55–58]. The increased glucose utilization found here at day 7 post SE further supports a (complex and) dynamic role of dysregulated glucometabolism during epilepsy development as well as its potential to serve as translational prognostic biomarker and target for metabolism-directed prophylactic intervention following epileptogenic brain insults [12]. Interestingly, increased glucose metabolism revealed with MMF in the present study was found in regions and at a time-point, for which we could show before that distinct microglia activation is also present [59]. This suggests a potential to increase sensitivity of ^18^F-FDG PET for imaging inflammation-associated changes in central nervous glucose metabolism by applying MMF anesthesia. Further studies will identify if premedication with a single component of MMF anesthesia might already increase sensitivity of ^18^F-FDG PET, which would also be of translational value.

To better understand changes in glucose metabolism—important for the establishment of new treatment options or early biomarkers—we aimed to establish standardized anesthesia protocols that can also be used for kinetic modeling. We found that MMF is most discriminative for hypermetabolic changes during epileptogenesis compared to other anesthesia protocols tested. Furthermore, in order to guarantee the best possible detection of changes additional SPM analysis and kinetic modeling is recommendable.

## Supporting information

S1 Fig Blood glucose levels. Averaged blood glucose levels resulting from two blood samples drawn before ^18^F-FDG injection and after the CT. Significant changes (P<0.05; one-way ANOVA with Dunnett’s multiple comparisons post hoc test) between BL levels are indicated by *. Differences between BL and blood glucose levels further scans of each anesthesia (indicated by #) were tested by one-way ANOVA and Tukey’s post hoc test or by one-way ANOVA with Dunnett’s multiple comparisons post hoc test. Data is presented as mean ± SD.
(TIF)

S2 Fig Regional 18F-FDG uptake is displayed for BL, 7 d post SE and the chronic epileptic phase under (A) awake uptake condition, (B) isoflurane, (C) MMF, and (D) propofol anesthesia. Data is presented as mean ± SD. Significant changes between BL uptake and following scans were tested by a one-way ANOVA followed by Dunnett’s post hoc test, * P<0.05.
(TIF)

S3 Fig Averaged coronal images of ^18^F-FDG uptake (30–60 minutes after tracer injection, -3.6 mm relative to bregma) are displayed for BL, 7 d post SE and chronic epilepsy for awake uptake condition, isoflurane, MMF and propofol anesthesia. (TIF)

S4 Fig Calculation of the metabolic rate of glucose MRGlu by a 2-tissue compartment model for (A) continuous isoflurane (B) MMF and (C) propofol anesthesia. Data is presented as mean ± SD. Significant changes in brain regions between BL and following scans were tested by one-way ANOVA followed by Dunnett’s multiple comparisons test, * indicates significant differences (P<0.05).
(TIF)

S1 Table Animal numbers at the different imaging time points. (PDF)

S1 File Anonymized data set. (PDF)
