# Supplementary figures and images for: Choice of anesthesia and data analysis method strongly increases sensitivity of 18F-FDG PET imaging during experimental epileptogenesis
Source: PLoS One. 2021 Nov 24;16(11):e0260482. doi: 10.1371/journal.pone.0260482 (PMC8612569; doi:10.1371/journal.pone.0260482)

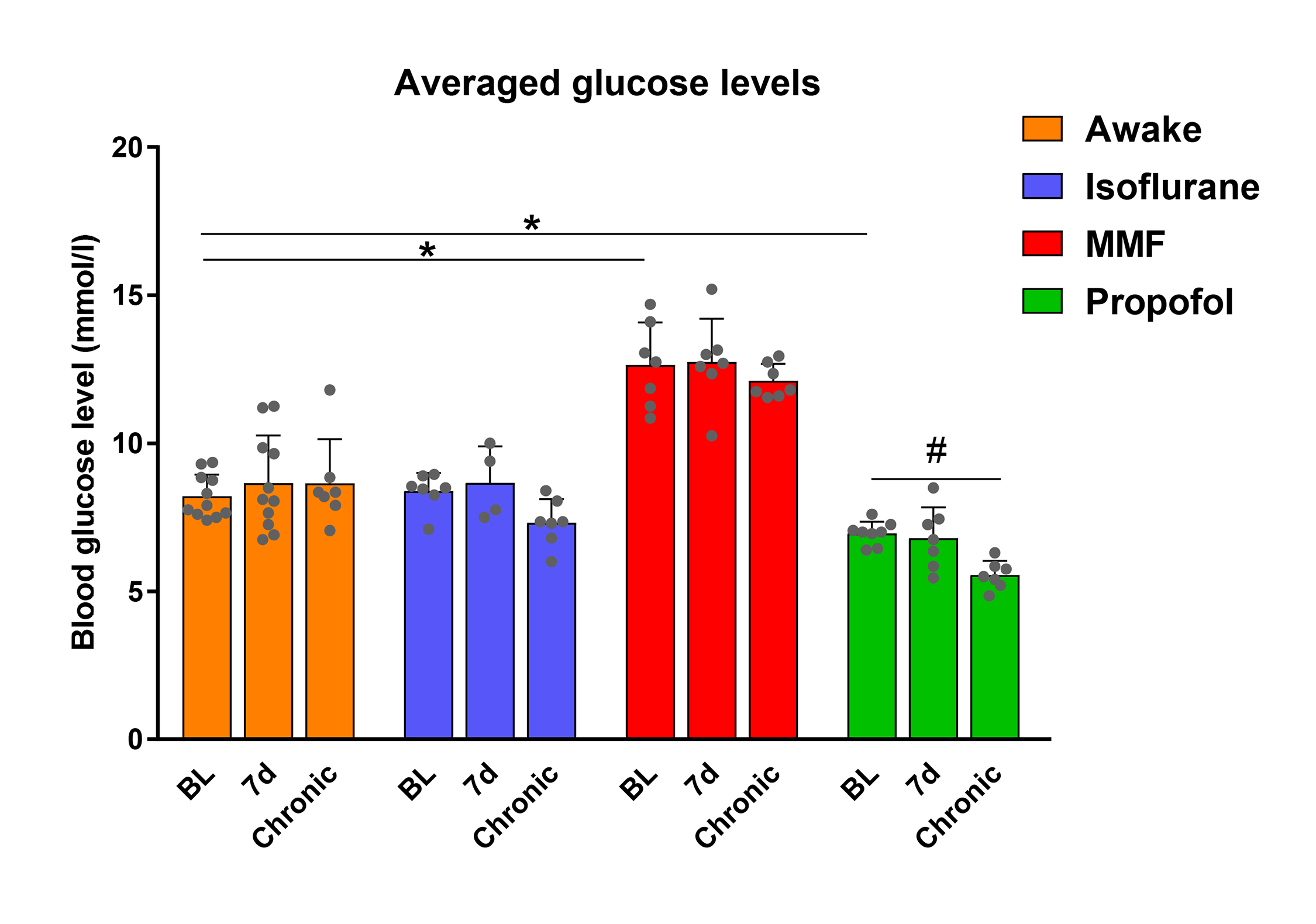

Supplement: S1 Fig — Averaged blood glucose levels resulting from two blood samples drawn before 18F-FDG injection and after the CT. Significant changes (P<0.05; one-way ANOVA with Dunnett’s multiple comparisons post hoc test) between BL levels are indicated by *. Differences between BL and blood glucose levels further scans of each anesthesia (indicated by #) were tested by one-way ANOVA and Tukey’s post hoc test or by one-way ANOVA with Dunnett’s multiple comparisons post hoc test. Data is presented as mean ± SD. (TIF) [file pone.0260482.s001.tif]

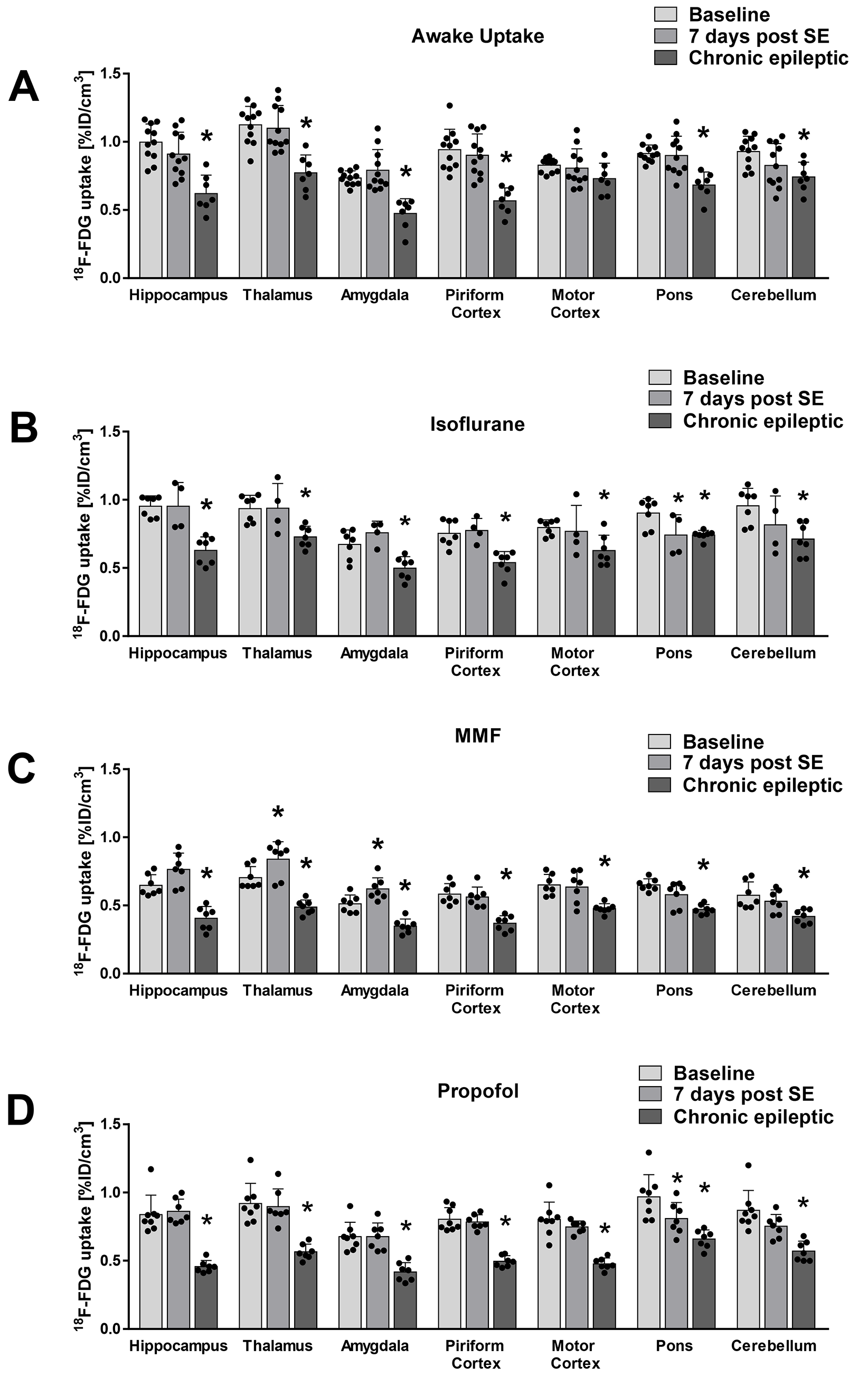

Supplement: S2 Fig — Regional 18F-FDG uptake is displayed for BL, 7 d post SE and the chronic epileptic phase under (A) awake uptake condition, (B) isoflurane, (C) MMF, and (D) propofol anesthesia. Data is presented as mean ± SD. Significant changes between BL uptake and following scans were tested by a one-way ANOVA followed by Dunnett’s post hoc test, * P<0.05. (TIF) [file pone.0260482.s002.tif]

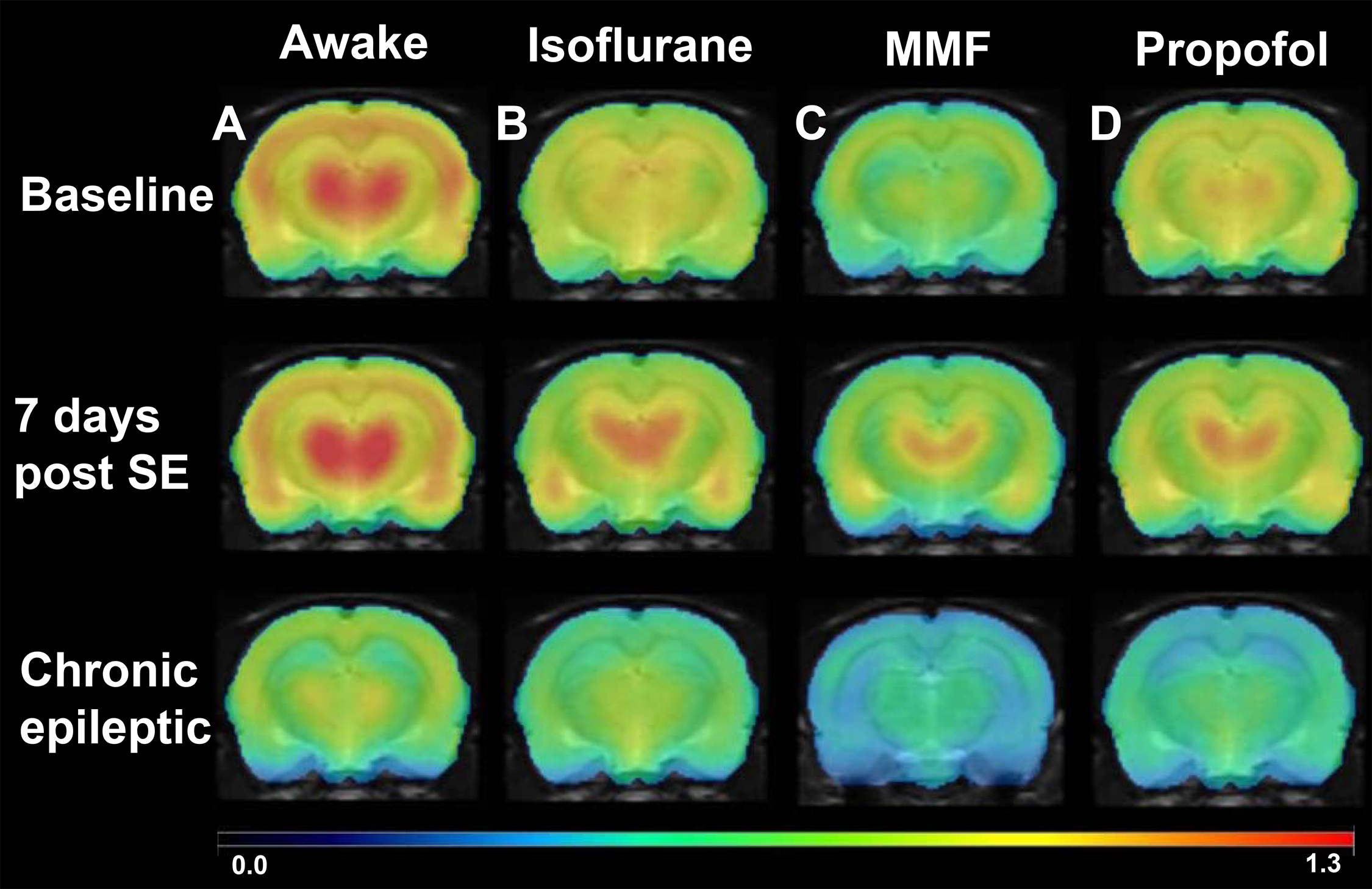

Supplement: S3 Fig — (TIF) [file pone.0260482.s003.tif]

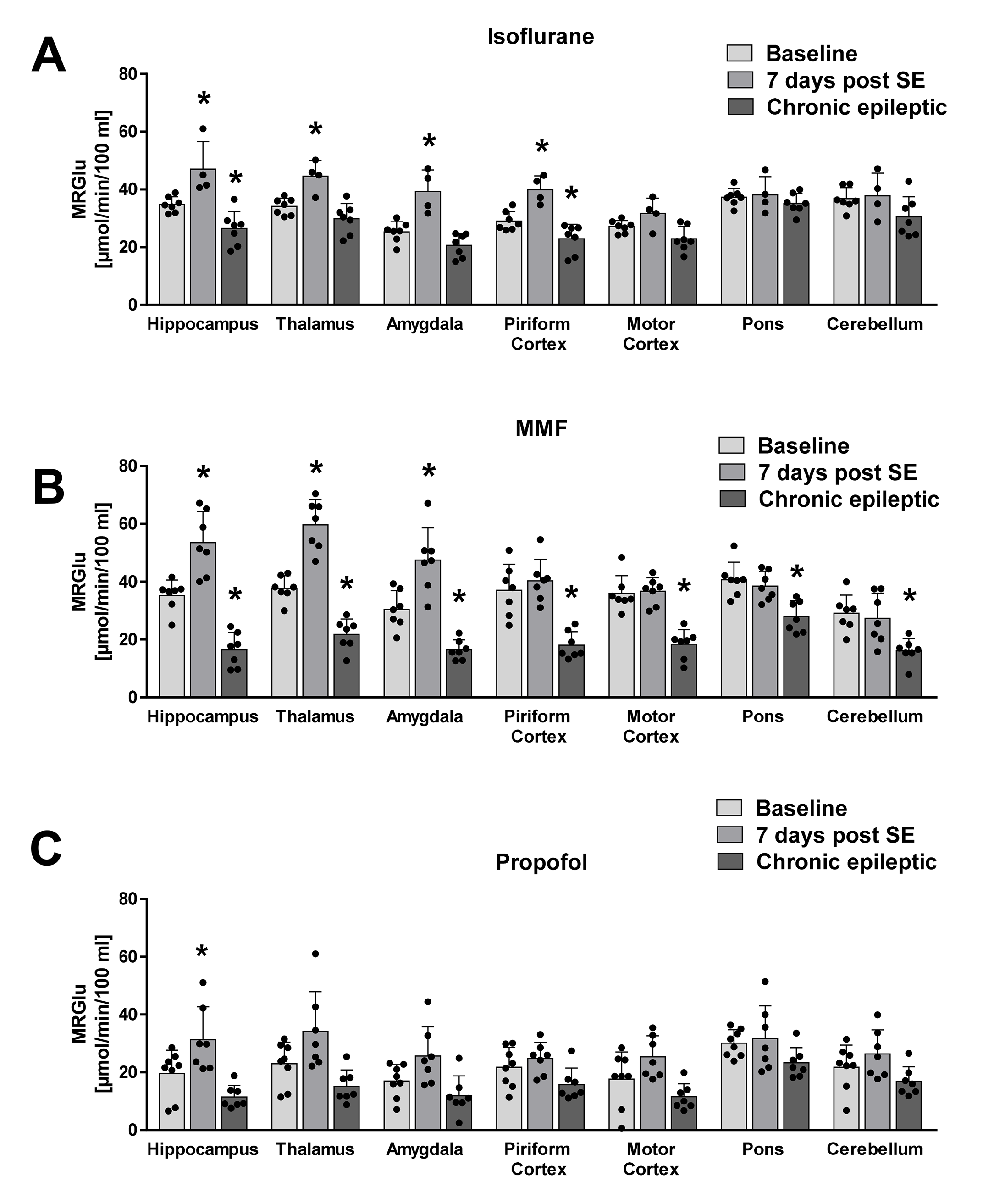

Supplement: S4 Fig — Calculation of the metabolic rate of glucose MRGlu by a 2-tissue compartment model for (A) continuous isoflurane (B) MMF and (C) propofol anesthesia. Data is presented as mean ± SD. Significant changes in brain regions between BL and following scans were tested by one-way ANOVA followed by Dunnett’s multiple comparisons test, * indicates significant differences (P<0.05). (TIF) [file pone.0260482.s004.tif]
