# Supplementary material for: Choice of anesthesia and data analysis method strongly increases sensitivity of 18F-FDG PET imaging during experimental epileptogenesis
Source: PLoS One. 2021 Nov 24;16(11):e0260482. doi: 10.1371/journal.pone.0260482 (PMC8612569; doi:10.1371/journal.pone.0260482)
Supplement: S1 Table — (PDF) [file pone.0260482.s005.pdf]

Supplemental Table 1. Animal numbers at the different imaging time points.

	Awake	Isoflurane	MMF	Propofol
Baseline	11	7	7	8
7d post SE	11	4	7	7
12-14 w post SE	7	7	7	7
