# Supplementary material for: Choice of anesthesia and data analysis method strongly increases sensitivity of 18F-FDG PET imaging during experimental epileptogenesis
Source: PLoS One. 2021 Nov 24;16(11):e0260482. doi: 10.1371/journal.pone.0260482 (PMC8612569; doi:10.1371/journal.pone.0260482)
Supplement: S1 File — (PDF) [file pone.0260482.s006.pdf]

Figure 2 Blood glucose levels

Values are given in mmol/l

Before the scan

Awake Baseline	Awake 7d post SE	Awake Chronic epileptic	Isoflurane Baseline	Isoflurane 7d post SE	Isoflurane Chronic epileptic	MMF Baseline	MMF 7d post SE	MMF Chronic epileptic	Propofol Baseline	Propofol 7d post SE	Propofol Chronic epileptic
6,2	7,6	6,6	8,5	6,4	6,1	9,3	9,4	7,3	6,7	6,6	6,3
6,9	7	5,9	7,6	6,6	5,2	9,8	6,7	7,1	6,7	5,6	7,2
7,3	7,7	6,3	7,2	7,4	6,2	6,7	6,1	7,3	5,2	5,9	5,8
8,1	6,3	7,4	6,8	5,4	6,8	7,5	6,6	6,3	5,7	5,9	5,7
6,4	5,6	7,4	6,7		6,4	7,7	9,5	7,4	6,8	7,7	5,8
7,4	6,2	5,9	6,1		6,3	8,8	8,1	6,2	5,4	8	6,1
6,5	6,9	7	6,5		6,3	8,5	7,7	6,8	7	4,8	5,4
8,1	6,2								6,7		
7,7	7,8										
6,8	5,7										
6,7	6										

At the end of the scan

Awake Baseline	Awake 7d post SE	Awake Chronic epileptic	Isoflurane Baseline	Isoflurane 7d post SE	Isoflurane Chronic epileptic	MMF Baseline	MMF 7d post SE	MMF Chronic epileptic	Propofol Baseline	Propofol 7d post SE	Propofol Chronic epileptic
8,2	17,9	9,8	9,4	8,6	10	12,4	15,3	15,9	8,5	7,9	6,3
9,7	12,7	10,8	10,2	12,2	6,8	16,3	13,8	18,4	7,2	6,1	4,3
11,3	9,3	10,4	9,7	12,6	7,4	15,8	20,2	18,6	9,3	7,6	4,6
9,4	7,2	8,4	10,3	10,1	7,8	16,2	19,4	19,6	7,1	6,8	5,3
8,6	5,4	16,2	9,8		10,4	17,8	20,9	16,2	7,3	7,2	5
10,3	7,6	8,2	10,9		8,4	20,6	17,1	17,3	7,5	9	5,6
8,8	9,2	10,7	7,7		8,4	19,7	17,7	16,3	7	6,1	4,3
7,7	9,1								7,3		
11	14,6										
8,7	8,8										
8,5	13,3										

Figure 3A FDG Uptake Baseline

Values are given in %ID/cm3

Awake

Hippocampus	Thalamus	Amygdala	Pir. Cort.	Mot. Cort.	Cerebellum
0,995333	1,183428	0,748682	0,979238	0,987272	1,001855
0,979174	1,208958	0,813807	1,025078	0,949804	0,937073
0,896824	1,003087	0,736059	0,882125	0,958368	0,871691
0,810426	0,992308	0,688629	0,827508	0,819073	0,766684
0,913025	1,054359	0,642477	0,801934	0,995275	0,81035
1,14772	1,310619	0,753713	1,00124	1,076561	0,954959
1,063337	1,109786	0,691448	0,813699	1,084291	0,960685
1,163194	1,266254	0,791332	1,05895	1,220225	1,072382
1,136801	1,202376	0,789006	1,26544	1,256981	1,022703
1,075621	1,174507	0,726332	0,971559	1,181398	1,057933
0,784555	0,856348	0,692841	0,739956	0,781903	0,757101

Isoflurane

Hippocampus	Thalamus	Amygdala	Pir. Cort.	Mot. Cort.	Cerebellum
1,017161	1,035005	0,78114	0,847967	0,82407	1,112587
1,00956	0,9974	0,771669	0,857912	0,839535	1,025096
0,897812	0,826504	0,55593	0,616763	0,733653	0,794299
0,856033	0,86349	0,654799	0,70385	0,770912	0,909897
1,006524	0,990871	0,708738	0,726322	0,849985	1,040901
1,017461	1,025748	0,730742	0,845936	0,843759	1,025141
0,862973	0,814569	0,506721	0,684298	0,714437	0,78061

MMF

Hippocampus	Thalamus	Amygdala	Pir. Cort.	Mot. Cort.	Cerebellum
0,554693	0,80707	0,537261	0,546796	0,733742	0,697557
0,531571	0,642651	0,443782	0,492792	0,625791	0,486985
0,704509	0,830247	0,620578	0,627027	0,762647	0,719495
0,616877	0,644511	0,518595	0,550371	0,563695	0,527679
0,528214	0,643006	0,465036	0,504317	0,570143	0,48623
0,654289	0,709953	0,549806	0,601667	0,680035	0,596341
0,495483	0,651316	0,445194	0,465967	0,618651	0,508974

Propofol

Hippocampus	Thalamus	Amygdala	Pir. Cort.	Mot. Cort.	Cerebellum
0,911603	0,969588	0,727585	0,793619	0,852876	0,9183
0,722589	0,771858	0,621974	0,626765	0,612634	0,717167
0,865764	0,897942	0,679995	0,702633	0,819969	0,786675
0,775912	0,884022	0,675754	0,707628	0,790318	0,825286
0,730449	0,787118	0,563045	0,614345	0,705802	0,7937
0,932844	1,238169	0,900692	0,929223	1,053114	1,199385
0,748883	0,956976	0,665102	0,678304	0,803185	0,871405
0,745359	0,853382	0,579461	0,615521	0,790727	0,845845

Figure 5A TAC Amygdala

Values are given in kBq/cm3/cm3

Isoflurane

Time [s]							
1	0	0	0	0	0	0	0
3	0	0	0	0	0	0	0
5	0	0	0	0	0	0	0
7	0	0	0	0	0	0	0
9	0	0	0	0	0	0	0
12,5	50,13	28,9	0,01	18,83	10,59	47,78	49,18
17,5	89,92	69,72	78,4	54,75	51,07	94,81	75,19
22,5	91,15	99,96	106,18	72,05	87,98	113,06	99,71
27,5	90,88	88,44	114,62	108,09	111,01	133,44	88,04
35	107,61	102,23	106,1	101,73	103,29	127,67	114,91
45	119,51	113,96	112,23	115,31	127,18	113,42	103,27
55	131,69	100,05	119,47	118,56	118,99	167,08	124,99
75	147,52	116,8	126,94	115,02	117,39	140,17	117,52
105	152,23	114,02	125,6	127,63	143,2	123,4	139,14
135	146,92	132,63	132,04	136,54	148,72	151,94	146,95
165	152,02	136,96	124,46	136,23	149,35	154,25	116,88
195	151,35	126,39	119,08	128,36	144,61	175,51	132,17
225	164,83	150,27	123,29	140,07	141,53	158,87	148,2
255	194,55	138,69	129,32	143,42	145,55	165,42	132,49
285	165,61	144,6	122,82	124,8	144,54	167,87	126,16
330	169,33	140,27	116,5	143,08	145,55	169,81	134,41
390	170,34	138,49	136,52	148,72	149,39	147,67	128,05
450	166,83	146,71	120,2	135,07	141,84	161,29	124,22
510	155,15	147,05	112,53	126,73	131,24	159,97	120,57
570	144,97	138,76	121,08	138,55	134,62	161,98	121,85
750	162,18	140,62	113,78	138,44	137,93	155,23	117,55
1050	157,91	131,4	104,93	134,06	139,52	155,44	107,32
1350	161,63	131,61	109,55	126,31	138,6	149,7	107,27
1650	155,95	133,92	104,12	130,01	140,15	149,18	111
2100	158,78	136,36	102,44	128,63	137,55	146,16	105,34
2700	155,04	135,4	106,39	131,79	139,22	142,97	108,96
3300	177,66	133,71	102,71	131,72	137,6	139,62	104,87

Propofol

0	0	0	0	0	0	0	0
0	0	0	0	0	0	0	0
0	0	0	0	0	0	0	0
92,68	16,01	0	0	0	0	0	0
89,23	95,02	9,81	61,03	96,27	0	114,93	1,63
73,41	110,42	103,3	99,59	104,79	80,29	123,94	131,78
73,02	104,2	114,87	74,15	83,22	75,3	133,94	123,69
67,18	114,63	102,38	67,5	79,28	82,1	125,48	110,07
83,15	84,54	107,02	87,63	101,64	80,29	108,07	106,08
88,69	99,64	107,27	93,97	114,35	94,71	123,08	138,69
82,29	129,58	120,32	103,39	111,69	90,89	137,72	111,47
81,36	118,64	126,99	115,21	98,24	105,43	130,63	138,32
89,76	129,88	156,75	123,93	118,02	103,03	140,8	126,48
109,1	131,86	179,88	118,94	104,15	113,2	140,06	138,32
98,56	136,37	189,31	127,85	125,29	128,57	149,94	153,18
106,8	137,04	178,56	129,75	119,13	125,17	170,75	166,17
124,55	156,37	158,11	140,73	119,26	130	147,15	136,44
103,69	159,96	179,64	147,34	122,5	128,01	161,17	155,62
105,83	142,52	180,03	129,63	134,86	123,51	157,64	162,58
109,01	158,48	166,76	142,04	122,43	125,45	162,11	157,05
113,17	161,67	171,48	143,59	120,21	133,54	164,49	163,37
111,02	170,11	177,62	141,55	130,52	133,83	165,13	158,82
109,36	155,79	178,1	147,05	128,04	125,43	164,98	170,21
109,75	162,59	177,09	135,46	128,52	133,97	162,41	158,08
111,52	163,67	172,86	138,68	128,78	130,87	167,94	151,52
111,82	157,86	176,02	129,4	121,15	125,12	153,66	149,57
108,22	158,79	168,05	138,1	123,13	119,19	153,25	147,57
102,57	155,85	165,49	126,05	117,13	117,67	154,23	141,12
112,94	145,06	160,37	124,16	112,11	116,19	154,18	138,26
105,04	149,27	159,53	123,98	116,41	119,84	154,98	138,16
105,1	146,18	154,89	121,92	116,41	119,17	145,27	137,85
98,05	144,06	155,57	120,5	113,12	119,49	145,86	131,85

MMF

1	0	0	0	0	0	0	0
3	0	0	0	0	0	0	0
5	0	0	0	0	0	0	0
7	0	0	0	0	0	0	0
9	0	0	0	0	0	0	0
12,5	0	43,28	0	0	19,18	0	0
17,5	64,68	84,23	57,6	68,69	59,35	55,05	61,91
22,5	106,83	96,1	89,6	123,7	77,08	96,36	86,23
27,5	103,8	105,53	83,84	113,6	79,42	98,7	87,04
35	105,84	97,32	86,88	115,06	85,67	88,74	83,99
45	90,93	83,31	101,61	94,26	89,42	93,87	91,24
55	93,62	80,83	104,86	113,15	96,19	93,08	114,4
75	102,48	92,45	117,55	116,69	95,37	96,78	98,21
105	111,19	105,92	127,92	128,74	118,74	98,29	110,57
135	119,99	113,76	144,22	137,86	121,63	114,47	133,6
165	126,03	120,05	147,33	148,61	127,45	124,46	131,01
195	113,03	121,16	157,06	149,36	127,14	129,85	122,26
225	113,44	126,03	159,05	137,77	122,35	121,96	130,57
255	116,03	128,29	151,27	134,85	124,59	122,59	125,12
285	124,19	114,4	149,7	146,7	133,45	119,37	131,92
330	126,95	115,62	144,8	132,5	136,94	123,39	133,06
390	119,18	119,37	165,89	139,86	128,12	127,7	139,14
450	122,01	111,56	150,26	141,88	130,41	122,22	127,74
510	119,68	115,74	140,44	145,85	132,64	123,44	119,96
570	102,39	116,99	143,29	127,93	123,15	118,38	123,52
750	108,45	106,42	146,68	129,05	112,68	118,98	118,05
1050	104,8	98,4	135,33	122,16	105,58	113,5	114,14
1350	96,88	98,52	137,63	116,7	104,23	106,46	105,53
1650	99,68	88,27	129,26	113,33	100,03	102,11	109,23
2100	101,75	87,14	127,98	106,71	95,09	103,62	104,6
2700	101,33	84	126,36	106,06	92,85	100,68	102,63
3300	96,18	82,19	120,62	98,32	88,51	95,99	99,14

Figure 5B TAC Thalamus

Values are given in kBq/cm3

Time [s]	Isoflurane							Propofol							
1	0	0	0	0	0	0	0	0	0	0	0	0	0	0	0
3	0	0	0	0	0	0	0	0	0	0	0	0	0	0	0
5	0	0	0	0	0	0	0	0	0	0	0	0	0	0	0
7	0	0	0	0	0	0	0	28,86	0,03	0	0	0	0	0	0
9	0	0	0	0	0	0	0	56,6	80,47	0,18	23,45	71,19	0	63,46	0,36
12,5	35,91	15,59	0,16	1,11	4,8	25,85	35,24	64,74	96,05	90,47	87,07	59,55	61,5	90,45	68,83
17,5	49,55	54,61	61,34	42	43,89	57,64	51,04	51,78	86,48	117,07	74,31	50,51	68,34	104,2	81,61
22,5	82,43	67,1	88,67	54,03	62,1	95,54	73,08	76,74	96,32	81,95	76,52	78,03	74,26	84,82	80,08
27,5	104	107,84	91,99	82,54	77,4	78,29	75,88	71,51	93,46	109,33	98,57	69,47	97,26	128,32	97,94
35	97,1	91,12	95,82	89,54	96,29	108,01	109,54	78,01	97,03	113,87	101,46	73,12	88,28	111,08	134,23
45	119,1	97,47	103,86	106,65	90,29	125,13	85,2	87,15	109,31	107,65	97,35	80,58	102,4	150,93	151,83
55	123,67	115,79	116,91	123,69	112,68	132,52	110,92	90,15	127,31	153,42	104,74	90,4	102,49	140,45	130,79
75	133,08	133,19	135,61	118,72	136,79	165,87	132,46	107,7	144,07	146,53	129,68	103,8	117,57	156,07	167,35
105	157,24	148,72	152,21	150,34	163,86	178,93	166,38	110,23	156,33	169,61	137,55	123,12	139,7	173,97	172,89
135	177,79	170,74	172,6	166,34	173,16	182,16	169,78	118,1	166,37	181,33	152,22	132,26	137,85	186,1	207,17
165	184,51	160,32	169,92	162,17	177,86	192,34	177,97	125,23	164,7	191,41	154,35	141,72	142,89	200,29	202,86
195	185,19	174,04	160,76	162,56	174,84	195,93	167,77	132,76	160,24	211,48	145,23	137,36	148,07	206,33	216,66
225	191,73	172,08	152,38	170,2	181,6	194,17	176,61	136,82	170,94	215,93	159,07	167,39	153,98	237,59	213,62
255	196,19	177,51	149,91	168,46	159,4	189,2	173,7	137,12	171,51	210,71	157,28	146,45	157,29	234,54	211,54
285	192,35	172,61	164,75	163,51	186,39	195,33	178,69	155,57	183,12	217,72	159,89	156,29	158,23	219,16	229,83
330	196,93	164,75	165,7	169,2	174,8	204,98	166,47	133,26	194,41	206,54	174,21	148,01	159,12	214,73	220,79
390	189,09	171,03	168,48	173,47	178,09	198,8	186,41	137,77	179,19	205,85	164,68	157,56	163,36	225,66	217,92
450	205,43	175,62	162,68	158,97	178,69	203,45	170,13	147,99	192,01	218,75	163,51	169,31	158,4	229,6	212,94
510	192,11	168,73	166,3	165,4	174,41	209,65	163,12	138,51	194,03	214,81	155,31	165,88	157,1	230,81	219,42
570	194,97	161,81	156,65	160,75	175,15	192,69	170,7	136,1	182,67	216,17	157,51	168,86	146,23	224,02	225,98
750	198,02	165,17	154,16	164,92	183,68	201,97	171,96	134,8	195,63	206,8	160,8	155,7	155,9	223,2	220
1050	202,02	169,18	152,1	168,85	185,49	198,29	166,69	134,07	184,89	209,56	160,7	156,67	151,59	217,95	215,67
1350	205,45	169,76	152,82	167,44	179,26	205,46	166,39	140,04	184,26	208,25	151,52	161,92	156,42	218,51	213,52
1650	204,78	174,54	149,82	166,81	183,97	207,67	165,26	142,36	185,53	205,47	155,82	159,36	158,14	216,07	208,29
2100	210,18	175,26	155,67	170,48	188,22	206,3	172,2	138,4	180,49	204,39	161,63	159,3	162,52	213,92	205,89
2700	209,41	172,67	154,18	167,61	187,69	203,06	169,41	134,93	182,47	212,59	157,57	162,03	163,03	216,22	199,09
3300	212,47	176,16	153,31	164,25	187,76	192,48	171,47	137,37	182,47	203,66	160,12	162,29	167,27	211,74	195,68

	MMF						
1	0	0	0	0	0	0	0
3	0	0	0	0	0	0	0
5	0	0	0	0	0	0	0
7	0	0	0	0	0	0	0
9	0	0	0	0	0	0	0
12,5	0	35,86	0	0	0,43	0	0
17,5	43,59	59,55	47,99	36,75	37,53	33,09	35,98
22,5	76,93	89,71	67,42	78,08	59,18	47,32	60,85
27,5	105,77	103,34	94,45	91,72	77,56	81,71	80,61
35	100,98	107,57	95,66	122,59	96,54	86,87	82,54
45	112,58	97,87	109	121,41	99,34	102,99	95,24
55	126,34	114,16	130,84	132,83	120,79	129,81	93,64
75	140,51	138,93	156,9	150,56	140,74	123,32	131,14
105	149,67	149,1	173,72	173,48	161,87	159,38	145,9
135	168,09	168,93	191,83	191,98	162,77	147,73	148,56
165	154,36	160,08	200,83	185,91	167,62	174,8	158,38
195	184,33	160,86	211,83	178,22	175,52	159,31	172,63
225	174,9	161,62	204,07	181,33	160,58	160,52	159,74
255	180,95	154,33	214,76	184,69	178,67	177,37	167,37
285	170,5	158,59	205,07	194,26	169,1	168,57	169,26
330	177,23	159,79	211,32	191,61	172,89	177,05	163,81
390	180,67	164,85	205,26	185,54	178,59	166,18	173,32
450	172,23	166,86	203,36	185,05	170,89	163,53	162,15
510	181,94	149,33	194,86	176,89	166,08	159,07	166,79
570	175,68	154,34	208,82	181,76	158,86	160,64	158,7
750	165,28	146,76	192,07	168,77	156,36	161,06	149,07
1050	159,8	143,36	185,48	160,36	148,63	149,08	141,63
1350	156,39	142,67	179,24	154,69	141,9	149,62	143,49
1650	158,19	134,3	181,58	146,79	136,28	142,31	136,99
2100	153,58	128,54	176,08	136,22	135,04	134,75	133,29
2700	151,72	122,57	165,9	131,21	127,64	129,09	126,15
3300	144,24	115,75	159,66	119,19	119,56	123,92	117,81

Figure 5B IDIF

Values are given in kBq/cm3

Time [s]	Isoflurane							Propofol							
1	0	0	0	0	0	0	0	0	0	0	0	0	0	0	0
3	0	0	0	0	0	0	0	0	0	0	0	0	0	0	0
5	0	0	0	0	0	0	0	0	0	0	0	0	0	0	0
7	0	0	0	0	0	0	0	124,21	0	0	0,04	320,14	0	201,21	0
9	0	0	0	0	0	0	0	264,66	129,74	288,55	181,85	386,21	3,97	306,78	181,08
12,5	109,97	74,1	0	0,51	0,08	114,71	62,93	185,73	277,79	209,2	316,53	299,9	278,8	393,23	331,82
17,5	229,4	266,31	195,44	222,5	164,57	285,43	205,13	145,68	278,92	106,44	250,54	236,93	271,86	361,76	350,98
22,5	263,93	342,07	297,45	299,47	219,59	375,92	237,61	141,78	186,81	90,36	204,62	256,54	214,63	248,33	267,55
27,5	219,12	299,79	273,46	368,21	249,26	356	214,99	129,64	179,11	77,95	177,47	221,91	163,76	225,99	209,37
35	256,96	283,17	256,44	358,02	222	291,32	247,9	137,79	190,62	103,17	167,52	223,95	146,14	249,5	207,29
45	240,37	245,25	237,15	304	234,58	270,13	219,02	131,18	195,2	101,92	148,8	193,39	149,26	243,25	228,16
55	229,74	229,93	221,57	244,84	212,31	271,59	222,83	126,49	166,77	96,65	172,1	215,55	152,86	209,51	250,45
75	215,81	211,29	199,54	233,75	198,18	223,14	223,93	127,87	185,35	100,79	166,78	196,11	150,8	216,13	227,71
105	244,54	212,89	180,38	211,95	185,63	213,74	216,79	132,03	198,09	90,62	177,43	218,01	160,83	211,25	204,49
135	222,72	184,56	154,91	212,1	186,1	197,34	193,66	113,02	170,37	116,43	142,97	174,6	148,13	214,78	207,19
165	218,65	174,8	152,25	204,48	159,53	184,13	159,08	134,88	119,34	93,3	137,49	184,38	135,94	210,4	226,84
195	204,42	158,85	147,63	193,05	148,42	186,74	182,93	118,9	90,62	46,91	136,3	181,18	139,98	193,35	206,51
225	168,87	163,91	129,73	172,98	169,75	183,25	164,52	130,76	103,28	72,7	145,2	174,19	122,79	198,17	200,71
255	192,14	154,53	128,64	177,43	140,74	177,58	150,03	123,58	95,12	66,89	142,13	174,57	154,7	178,79	190,2
285	175,01	155,07	126,99	164,1	130,49	167,4	145,97	107,58	75,54	64,02	125,85	168,74	131,43	207,52	200,47
330	173,04	142,84	122,62	147,63	139,37	153,52	140,04	106,12	82,86	69,56	126,54	171,98	132,77	176,23	197,36
390	166,78	131,92	117,1	152,36	142,21	141,97	141,49	108,92	87,82	66,51	123,28	159,01	147,61	187,12	189,27
450	150,76	130,61	108,37	154,82	121,11	143,84	138,63	105,61	89,1	81,7	117,5	157,65	119,51	164,02	172,5
510	141,05	123,12	103,96	137,28	122,76	150,7	121,37	98,72	83,71	72,74	102,83	139,72	121,36	168,61	162,93
570	144,59	123,83	100,28	132,41	124,79	129,72	126,87	82,83	98,69	92,33	114,06	138,66	114,55	148,71	167,29
750	131,59	109,51	91,41	116,27	104,91	120,73	103,79	88,94	87,18	61,13	93,99	127,65	104,88	146,75	141,14
1050	115,77	93,01	76,04	101,62	96,26	110,31	86,8	76,98	89,14	68,95	80,54	109,79	90,91	107,4	124,45
1350	105,15	84,11	75,85	92,61	94,25	95,44	78,06	69,23	82,52	69,99	70,94	97,05	86,17	108,35	108,62
1650	99,13	71,09	63,13	86,28	88,3	89,2	68,72	60,26	81,68	81,11	70,26	85,6	78,35	107,66	101,35
2100	89,53	68,05	58,06	72,34	77,31	76,01	63,61	56,65	87,32	76,04	54	81,84	66,44	92,57	91,36
2700	78,37	56,67	48,82	59,12	72,67	72,66	55	50,12	92,08	73,62	48,28	76,15	60,09	83,03	83,08
3300	75,02	47,77	40,23	49,75	69,33	57,4	50,57	45,14	88,89	73,11	44,48	66,56	51,71	73,14	71,33

	MMF						
1							
3	0	0	0	0	0	0	0
5	0	0	0	0	0	0	0
7	0	0	0	0	0	0	0
9	0	0	0	0	0	0	0
12,5	0	0	0	0	0	0	0
17,5	39,29	211,37	4,67	0,02	95,65	0	0
22,5	226,05	260,29	162,81	157,24	112,76	122,02	116,98
27,5	318,59	168,85	250,99	257	131,63	148,05	123,34
35	333,49	127,5	242,76	200,62	95,68	146,97	130,27
45	247,1	100,38	185,1	166,48	99,71	100,21	71,59
55	170,65	91,51	168,3	124,82	92,84	62,06	61,96
75	161,63	95,25	171,76	117,89	92,61	71,23	63,01
105	150,12	109,79	185,27	128,44	93,42	103,54	77,62
135	151,32	97,66	166,78	120,01	94,34	80,42	82,15
165	132,11	91,97	163,71	100,38	94,53	76,41	85,31
195	153,71	97,23	167,63	127,86	93,15	82,26	76,09
225	132,87	107,7	167,95	115,71	95,26	80,69	78,18
255	133,32	93,51	142,34	113,8	116,66	57,43	70,47
285	120,18	99,3	142,68	118,5	91,25	59,22	75,41
330	138,35	96,84	153,87	105,74	90,53	79,31	74,26
390	135,91	83,57	129,37	102,41	90,83	79,43	74,62
450	124,75	87,92	140,57	99,06	93,01	74,3	76,14
510	120,84	83,98	121,46	97,57	86,1	78,2	75,11
570	113,59	89,67	132,72	99,05	83,75	87,93	78,35
750	106,85	90,25	124,11	89,66	77,9	68,87	69,74
1050	93,03	77,7	105,82	75,9	76,48	74,15	67,79
1350	83,25	73,22	91,14	63,45	70,54	68,22	59,54
1650	70,68	66,48	76,22	55,34	65,86	63,3	49,11
2100	63,88	62,63	76,64	47,7	63,46	63,11	47,75
2700	57,83	64,02	64,97	43,34	55,56	63,6	44,78
3300	51,27	60,95	58,06	39,42	49,61	60,91	37,16
	47,32	54,58	55,82	33,76	51,9	58,95	36,77

Figure 6A Ki Isoflurane

Values are given in ml/g/min

Baseline

Hippocampus	Thalamus	Amygdala	Pir. Cort.	Mot. Cort.	Pons	Cerebellum
0,0291142	0,02989663	0,02281912	0,02664365	0,02285173	0,03054883	0,03454867
0,03219144	0,03146367	0,02505228	0,02874766	0,02504204	0,03518163	0,03465408
0,03269562	0,03151305	0,02232233	0,02345253	0,02389544	0,03248151	0,03047512
0,02718016	0,02772067	0,0224746	0,0241422	0,02492864	0,03279537	0,0310469
0,02855175	0,02730039	0,0204581	0,02317744	0,02389733	0,031776	0,03180424
0,03002924	0,03018053	0,02214853	0,02569823	0,02102646	0,03346153	0,03160093
0,0353599	0,0321619	0,01989239	0,02728395	0,0256332	0,0338556	0,03208231

7d post SE

Hippocampus	Thalamus	Amygdala	Pir. Cort.	Mot. Cort.	Pons	Cerebellum
0,0399871	0,0442608	0,03127709	0,03660494	0,03687589	0,03771637	0,03965746
0,04792167	0,03942986	0,03449847	0,03366739	0,0248698	0,03670196	0,03705949
0,03323763	0,03393304	0,03488316	0,03314339	0,02435614	0,02630749	0,02579821
0,03951865	0,03539364	0,03276187	0,03302586	0,02349386	0,03033674	0,02741561

12-14 w post SE

Hippocampus	Thalamus	Amygdala	Pir. Cort.	Mot. Cort.	Pons	Cerebellum
0,02558754	0,03019456	0,02266886	0,02438065	0,0207703	0,03558181	0,03201369
0,03588176	0,03944136	0,02274721	0,02882652	0,03458892	0,04149359	0,04116633
0,02986496	0,03192181	0,0236551	0,02660118	0,02383037	0,03821709	0,03098991
0,03695924	0,03812273	0,02484941	0,02780671	0,02912031	0,04044821	0,04324791
0,02180509	0,02672683	0,01415095	0,01346529	0,01762114	0,02943106	0,02242097
0,02040203	0,02410823	0,02388835	0,02680751	0,02209098	0,03566766	0,02396293
0,01871475	0,02234956	0,01508644	0,01658689	0,01676207	0,02956185	0,02449436

Figure 6B Ki MMF

Values are given in ml/g/min

Baseline

Hippocampus	Thalamus	Amygdala	Pir. Cort.	Mot. Cort.	Pons	Cerebellum
0,02194625	0,02599357	0,01774297	0,02423902	0,02309129	0,02418512	0,02156302
0,01413502	0,01694678	0,01164063	0,01408645	0,01624469	0,01875166	0,01129596
0,02457089	0,02502091	0,01988363	0,02441872	0,02504135	0,02648774	0,02004712
0,02320215	0,02679189	0,02448818	0,01978791	0,02122202	0,03263616	0,01893556
0,02112337	0,02110644	0,01564875	0,01726808	0,01942976	0,02353327	0,01457773
0,01853292	0,01812483	0,01582398	0,01623496	0,01761411	0,0204763	0,01315779
0,02176584	0,02212949	0,01969796	0,02251508	0,02533042	0,02202994	0,02091875

7d post SE

Hippocampus	Thalamus	Amygdala	Pir. Cort.	Mot. Cort.	Pons	Cerebellum
0,0307113	0,0395683	0,02830011	0,02282472	0,01863295	0,02029209	0,00948173
0,02885854	0,03905994	0,03352419	0,02966757	0,0271589	0,02561033	0,01840021
0,03663622	0,03475586	0,02175053	0,01922265	0,02144073	0,02295747	0,01840897
0,0285326	0,02976733	0,0177651	0,01763583	0,02090015	0,02140293	0,01147478
0,0326318	0,03422707	0,03260931	0,02649181	0,01935706	0,02185928	0,0182343
0,02424373	0,0275518	0,02976561	0,02373332	0,01746672	0,01881042	0,01284491
0,03422174	0,03833919	0,02944375	0,024716	0,02511603	0,02577617	0,0217978

12-14 w post SE

Hippocampus	Thalamus	Amygdala	Pir. Cort.	Mot. Cort.	Pons	Cerebellum
0,00828139	0,01201584	0,00996544	0,00940043	0,01224132	0,01433417	0,00972911
0,01062663	0,01431654	0,01288036	0,01468346	0,01161286	0,02021175	0,00958624
0,0128382	0,01433514	0,00891831	0,01091232	0,01119123	0,01890109	0,01038182
0,01397154	0,01630481	0,01100388	0,01328543	0,01454761	0,01836295	0,01263638
0,01111455	0,01479807	0,00801993	0,00826119	0,01295161	0,01506429	0,01066773
0,0060389	0,01178234	0,01058748	0,0095876	0,00828907	0,01688473	0,00964446
0,00603112	0,00813985	0,00811247	0,0097472	0,00653879	0,01402022	0,00503656

Figure 6C Ki Propofol

Values are given in ml/g/min

Baseline

Hippocampus	Thalamus	Amygdala	Pir. Cort.	Mot. Cort.	Pons	Cerebellum
0,02295728	0,02856653	0,02247183	0,02927124	0,02363031	0,03531892	0,02624794
0,00818305	0,01322144	0,01159839	0,0160216	0,00753069	0,02769908	0,01612278
0,00673	0,01164891	0,00730746	0,0115738	0,000693	0,02433522	0,00694423
0,02945247	0,03290932	0,02428013	0,03022662	0,02843757	0,03848874	0,02989759
0,02167382	0,02269116	0,01708728	0,02419137	0,01951247	0,02708463	0,02429933
0,02563624	0,02725391	0,01820667	0,01951735	0,02131498	0,03291187	0,02567217
0,02285373	0,02597593	0,01960376	0,0225417	0,01964888	0,03333158	0,02353892
0,03012027	0,03332859	0,02400773	0,03149131	0,03007701	0,03694021	0,03315025

7d post SE

Hippocampus	Thalamus	Amygdala	Pir. Cort.	Mot. Cort.	Pons	Cerebellum
0,0303128	0,03518488	0,02450681	0,02912601	0,03342067	0,0490225	0,03430302
0,02712461	0,02963576	0,02645769	0,02996829	0,0240943	0,02741584	0,02491572
0,02320286	0,02767478	0,01707346	0,02571044	0,01927687	0,02699415	0,02094397
0,03475424	0,02578045	0,01894061	0,03032745	0,02266763	0,02356013	0,02057019
0,0429858	0,04234513	0,03377601	0,03473173	0,03157094	0,03960754	0,02855333
0,02052244	0,02577228	0,02209442	0,03977105	0,02036577	0,0246948	0,02209967
0,02354162	0,02960501	0,02048049	0,01966077	0,01477611	0,02511853	0,01720593

12-14 w post SE

Hippocampus	Thalamus	Amygdala	Pir. Cort.	Mot. Cort.	Pons	Cerebellum
0,01083498	0,01455085	0,01289684	0,01525455	0,01173477	0,02541661	0,01556906
0,01167485	0,01499567	0,00320331	0,01536377	0,01103651	0,02682814	0,02050692
0,01071398	0,01254488	0,01339086	0,01815822	0,00960952	0,02593119	0,01684397
0,01798961	0,0238591	0,01970133	0,02358414	0,01886319	0,03254791	0,02661321
0,01875065	0,02484234	0,01557643	0,02275604	0,01765899	0,03498709	0,02342379
0,02374777	0,03211221	0,03146889	0,03465772	0,0251584	0,04239393	0,03355066
0,01338617	0,01759896	0,01446174	0,01670664	0,01277745	0,0280702	0,020183
